# The ω Subunit of RNA Polymerase Is Essential for Thermal Acclimation of the Cyanobacterium *Synechocystis* Sp. PCC 6803

**DOI:** 10.1371/journal.pone.0112599

**Published:** 2014-11-11

**Authors:** Liisa Gunnelius, Juha Kurkela, Kaisa Hakkila, Satu Koskinen, Marjaana Parikainen, Taina Tyystjärvi

**Affiliations:** Department of Biochemistry, University of Turku, Turku, Finland; CEA-Saclay, France

## Abstract

The *rpoZ* gene encodes the small ω subunit of RNA polymerase. A ΔrpoZ strain of the cyanobacterium *Synechocystis* sp. PCC 6803 grew well in standard conditions (constant illumination at 40 µmol photons m^−2^ s^−1^; 32°C; ambient CO_2_) but was heat sensitive and died at 40°C. In the control strain, 71 genes were at least two-fold up-regulated and 91 genes down-regulated after a 24-h treatment at 40°C, while in ΔrpoZ 394 genes responded to heat. Only 62 of these heat-responsive genes were similarly regulated in both strains, and 80% of heat-responsive genes were unique for ΔrpoZ. The RNA polymerase core and the primary σ factor SigA were down-regulated in the control strain at 40°C but not in ΔrpoZ. In accordance with reduced RNA polymerase content, the total RNA content of mild-heat-stress-treated cells was lower in the control strain than in ΔrpoZ. Light-saturated photosynthetic activity decreased more in ΔrpoZ than in the control strain upon mild heat stress. The amounts of photosystem II and rubisco decreased at 40°C in both strains while PSI and the phycobilisome antenna protein allophycocyanin remained at the same level as in standard conditions. The phycobilisome rod proteins, phycocyanins, diminished during the heat treatment in ΔrpoZ but not in the control strain, and the *nblA1* and *nblA2* genes (encode NblA proteins required for phycobilisome degradation) were up-regulated only in ΔrpoZ. Our results show that the ω subunit of RNAP is essential in heat stress because it is required for heat acclimation of diverse cellular processes.

## Introduction

DNA-dependent RNA polymerases (RNAPs) catalyze the transcription of genetic information from DNA to RNA. The core of the multi-subunit RNAP is conserved throughout all cellular life forms [1]. The RNAP core of the majority of eubacteria, contains a catalytic center consisting of β and β′ subunits [2], two identical α subunits that enhance transcription efficiency and participate in promoter recognition [3], and a small ω subunit. In cyanobacteria, however, the RNAP core consists of six subunits because β′ has been split into two parts, an N-terminal γ subunit and a C-terminal β′ subunit [4]. For promoter recognition and transcription initiation, the bacterial RNAP core recruits a σ factor. Bacteria encode one essential primary σ factor and varying number non-essential σ factors [5]. Different σ factors favor different promoters thus orchestrating the transcriptional efficiencies of different genes.

The ω subunit of the RNAP core is encoded by the *rpoZ* gene. Knock out strains of the ω subunit have been constructed in the proteobacterium *Escherichia coli* [6], the actinobacteria *Mycobacterium smegmatis* [7], *Streptomyces coelicolor* [8] and *Streptomyces kasugaensis* [9], and in the cyanobacterium *Synechocystis* sp. PCC 6803 [10], indicating that *rpoZ* is not an essential gene. Studies in *E. coli* have revealed that the ω subunit acts as a molecular chaperone for the β′ subunit [11], suggesting that the ω subunit has a similar role as the essential eukaryotic RPB6 subunit of RNAP [12]. We have recently shown that in the ΔrpoZ strain of *Synechocystis*, recruitment of the primary σ factor, SigA, by the RNAP core occurs less frequently than in the control strain, and as a consequence, many highly expressed genes are down-regulated in ΔrpoZ [10].

The optimum temperature for *Synechocystis* is 30–32°C but cells grow for a few days even at 43°C [13]–[15]. Pretreatment of *Synechocystis* cells in mild heat stress leads to acquired thermotolerance allowing survival in otherwise lethal temperatures up to 50°C [16]–[18]. Photosynthesis is a heat-sensitive process [19], and photosystem II (PSII) is the most vulnerable component, for which it takes hours to fully acclimate to an elevated temperature [20]. Transcriptomics and proteomics studies have revealed that heat treatment induces expression of many heat shock genes and numerous genes with unknown functions [20], [21].

Previous studies have shown that group 2 σ factors play roles in acclimation to elevated temperatures. The group 2 σ factor gene *sigB* is rapidly up-regulated upon a heat shock [22], [23] and the SigB factor, in turn, up-regulates especially the expression of the small heat shock protein HspA [14] and some other heat shock proteins [24]. Although SigC does not regulate heat shock genes, it is essential for heat acclimation processes as it is important for sustained functional photosynthesis in elevated temperatures [15], [25]. Upstream of the σ factors in the signaling cascades are histidine kinases (Hiks). For heat stress, Hik34 has been recognized as an important regulator, negatively controlling the expression of some heat shock genes like the *htpG* gene and the *groESL1* operon [26]. Furthermore the CIRCE/HrcA system has been shown to regulate the expression of some heat shock genes including the *groESL1* operon and the *groEL2* gene [27].

In the present study, the ω subunit of the RNAP core was found to be essential for the survival of cells even under mild heat stress. The results show that mild heat treatment at 40°C induces decrease of the RNAP content in the control strain but not in the ΔrpoZ strain. Furthermore, twice as many genes responded to heat treatment in ΔrpoZ than in the control strain (CS), and 80% of the heat-responsive genes were unique to ΔrpoZ. Mild heat stress induced reduction of light-saturated photosynthetic activity in both strains but this reduction was more prominent in ΔrpoZ than in CS. According to our results, many aspects of heat acclimation occurred differently in ΔrpoZ than in CS, and a combination of inappropriate responses in several cellular functions, rather than a deficiency in the expression of a single gene or operon, was the reason for the heat lethal phenotype of ΔrpoZ.

## Results and Discussion

### The ΔrpoZ strain has difficulties in acclimation to elevated temperature

**Figure 1:**
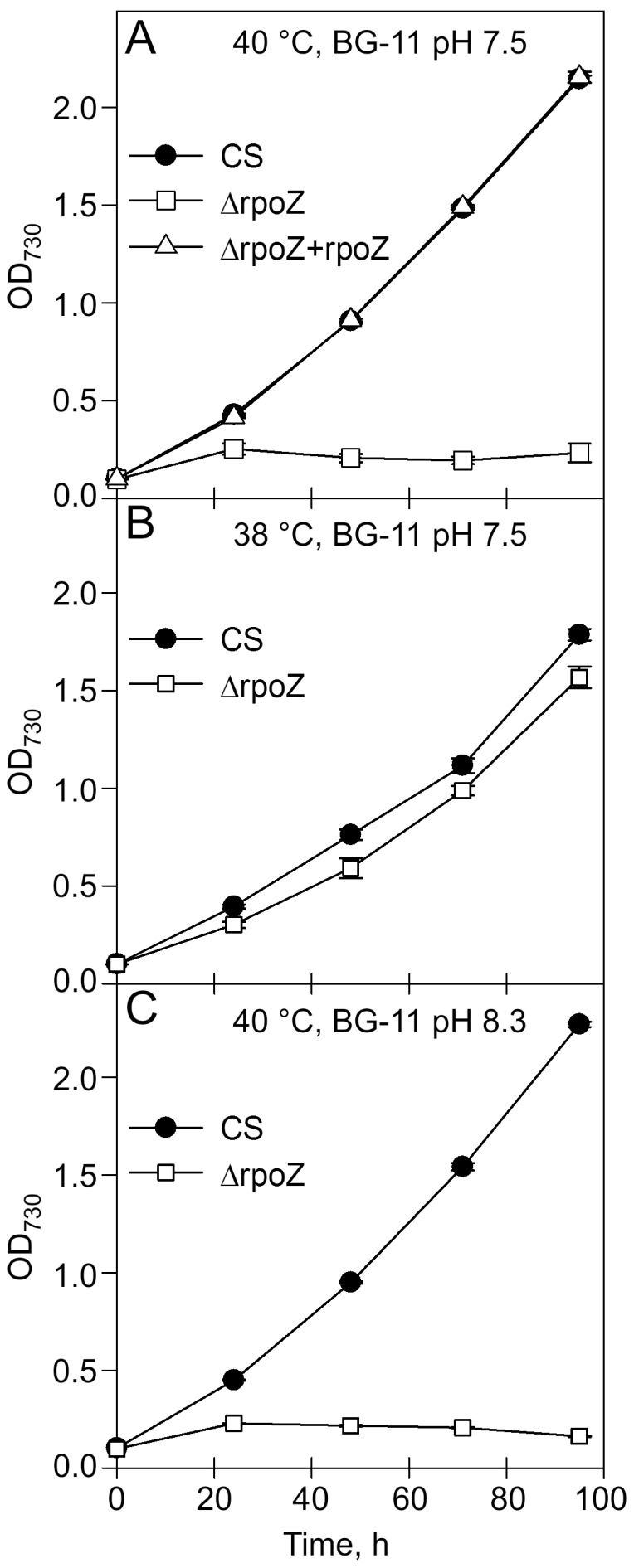
Growth of the control strain (CS; black circles), the ΔrpoZ strain (white squares), and a complementation strain ΔrpoZ+rpoZ (white triangles) in mild heat stress. Cells were grown at 40°C under continuous illumination of 40 µmol photons m^−2^ s^−1^ in BG-11 medium buffered with 20 mM Hepes, pH 7.5 (A), at 38°C, pH 7.5 (B) or at 40°C, pH 8.3 (C). At least three independent biological replicates were measured, and the error bars, shown if larger than the symbol, denote standard error of the mean (SEM).

In our standard growth conditions, continuous light at the photosynthetic photon flux density (PPFD) of 40 µmol m^−2^ s^−1^, and 32°C, the ΔrpoZ strain grows like CS [10]. At 40°C, CS grows essentially like it grows at 32°C (Fig. 1A), the doubling times during the first day being 11.6±0.2 h (Fig. 1) and 11.4±0.3 h [10] at 40°C and 32°C, respectively. The ΔrpoZ strain grew more slowly than CS during the first day at 40°C (Fig. 1A), with a doubling time of 18.5±2.0 h. A survival test indicated that the ΔrpoZ strain contained only 3.5×10^2^±0.4×10^2^ colony forming units (CFUs) after 24-h growth at 40°C while CS contained almost a hundred thousand times more CFUs, 3.4×10^7^±0.1×10^7^. Transfer of cells back to the standard conditions did not rescue ΔrpoZ cells after two days of incubation at 40°C, but cells died. The initial growth of ΔrpoZ was slow at 38°C, with the doubling times for the first day of 12.1±0.3 h and 15.2±0.8 h for CS and ΔrpoZ, respectively (Fig. 1B). At 38°C, however, the ΔrpoZ cells were able to acclimate, and similar doubling times, 25.4±0.5 h for CS and 25.2±1.6 h for ΔrpoZ, were measured after the second day (Fig. 1B). The ΔrpoZ+rpoZ strain, in which the *rpoZ* gene has been re-introduced to the genome under the strong *psbA2* promoter [10], grew similarly as CS at 40°C (Fig. 1A). This indicates that the heat-sensitive phenotype of ΔrpoZ is due to the lack of the ω subunit.

A DNA microarray analysis in standard conditions revealed that many genes involved in carbon concentrating mechanisms (CCM) and carbon fixation are down-regulated in the ΔrpoZ strain compared to CS [10]. Because temperature rise decreases the availability of inorganic carbon (the equilibrium concentration of dissolved CO_2_ at 40°C is only 82% of that at 32°C), we tested if growth can be rescued by improving the availability of soluble inorganic carbon by increasing the pH of the growth medium to 8.3. Alkaline conditions have been previously shown to rescue many mutants with deficiencies in carbon metabolism. The growth of the heat-sensitive σ factor mutant ΔsigC can be rescued by improving the availability of soluble inorganic carbon at 43°C by rising the pH of the growth medium from 7.5 to 8.3 [15], [25]. Furthermore, *Synechocystis* strains ΔNdhB, lacking a functional NAD(P)H dehydrogenase complex, and ΔNdhD3/NdhD4, with an inactivated CO_2_ uptake system, are able to grow at pH 8.3, but not at pH 7.5 [28], and even a mutant deficient of the main carboxysome operon can be grown in alkaline conditions [29]. In contrast to mutants with deficiencies in carbon concentrating mechanisms, the growth of ΔrpoZ cells at 40°C was not rescued at pH 8.3 (Fig. 1C), indicating that the heat-lethal phenotype of ΔrpoZ is probably not only caused by deficiencies in CCM. The growth rates of CS and ΔrpoZ were 10.6±0.2 h and 11.2±0.3 h, respectively, when cells were grown in BG-11 medium without added bicarbonate in standard conditions, confirming that ΔrpoZ cells are able to cope with low carbon conditions. Furthermore, the similarity of the growth rates in the presence and absence of added bicarbonate suggest that the bicarbonate addition to BG-11 has a negligible effect on the inorganic carbon content of the medium in growth experiments performed under ambient air.

Since ΔrpoZ survived only for a limited time at 40°C, all subsequent experiments were done by growing cells first in standard conditions to OD_730_∼1, and then transferring the cells to 40°C for 24 h. The 24-h heat treatment was selected because drastic difference between growth of mutant and CS was obvious after the first 24-h (Fig. 1A). Both strains grew during the 24-h treatment at 40°C (OD_730_ increased from 1.0 to 1.5 in CS and to 1.4 in ΔrpoZ, respectively), suggesting that dense ΔrpoZ cultures might tolerate high temperature better than dilute cultures.

### Comparison of gene expression of the control and ΔrpoZ strains at 40°C

**Figure 2:**
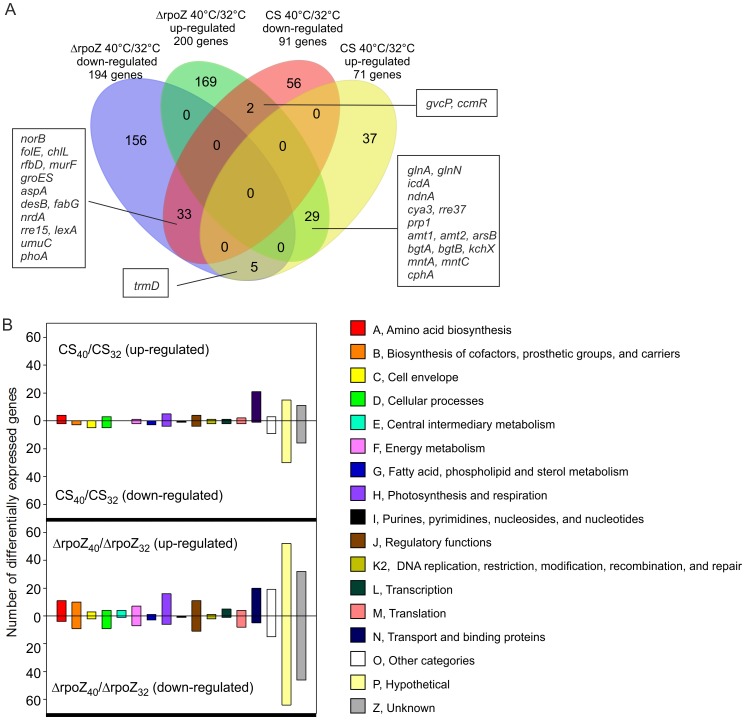
Genes responding to mild heat stress in the control (CS) or ΔrpoZ strains. (A) The Venn diagram shows genes down-regulated or up-regulated in ΔrpoZ or CS upon 24-h treatment at 40°C. The gene was considered as differently regulated if log_2_ of the fold change was ≤−1 or ≥1 and the P value was <0.05. The numbers inside the sectors indicate the numbers of overlapping and unique genes in different pairwise comparisons. Genes with known function that are down-regulated or up-regulated in both strains upon heat treatment are indicated, and also genes showing opposite response to heat treatment in the studied strains are included if their function is known. (B) Distribution of mild heat stress responsive genes to functional categories according to Cyanobase.

To get a more comprehensive picture on why ΔrpoZ is not able to acclimate to mild heat stress, gene expression changes were studied by DNA microarray analysis. For DNA microarray analysis, CS and ΔrpoZ were grown in standard conditions and then treated at 40°C for 24 h before RNA was isolated. In addition, the results from standard growth conditions [10] were used as controls. All microarray data are available in GEO (accessions GSE59451). In the control strain, 71 genes were at least two-fold up-regulated upon heat treatment and 91 genes were down-regulated to one half or less (Fig. 2A). Complete lists of up-regulated (Table S1) and down-regulated (Table S2) genes in CS are included as supplemental material. In ΔrpoZ, the heat treatment induced up-regulation of 200 genes (Fig. 2A, Table S3) and down-regulation of 194 genes (Fig. 2A, Table S4). Thus, 2.4 times more genes responded to mild heat treatment in the mutant strain than in CS (Fig. 2A).

**Figure 3:**
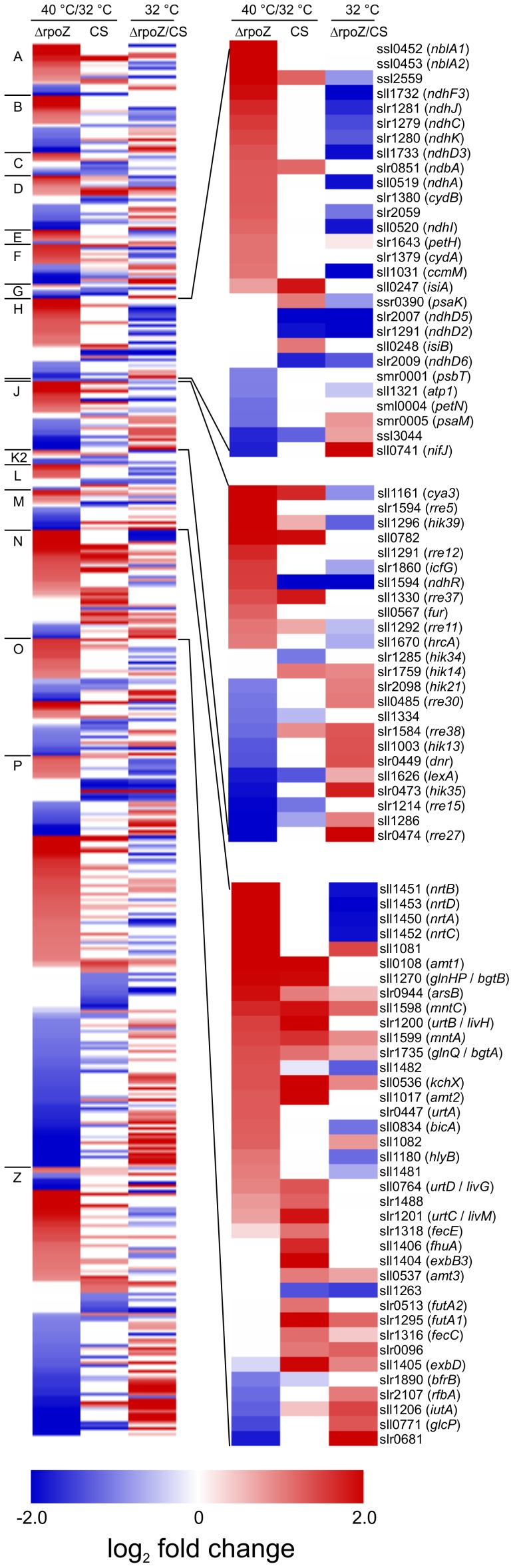
Comparison of heat stress responsive genes in CS and ΔrpoZ. The left panel shows genes whose expression was at least two-fold up-regulated or down-regulated either in the control or ΔrpoZ strains or both upon heat treatment when the gene expression was compared to the expression of the same strain under standard growth conditions, and in addition gene expression of ΔrpoZ and CS were compared in standard growth conditions. The heat maps show log_2_ fold change values (P<0.05) on the scale from −2 (blue) to 2 (red); values bigger than 2 are also shown in red and values smaller than −2 are blue. If the P value was ≥0.05, the fold change was given the value 0. Genes were arranged to categories according to Cyanobase, letters on the left indicating the same categories as in Fig. 2B. On the right, magnification of differently regulated genes in photosynthesis (top), regulatory functions (middle), and transport and binding proteins (bottom) is shown.

The differentially expressed genes were assigned to functional categories according to Cyanobase (Fig. 2B), and a heat map was constructed to further facilitate comparison between strains (Fig. 3). The heat map includes genes that were up or down regulated upon mild heat stress in ΔrpoZ, in CS or both, and in addition transcript levels of these heat-responsive genes were compared in ΔrpoZ and CS in standard growth conditions. All results included in Fig. 3 are collected in Table S5.

Only 33 genes were down-regulated upon mild heat-treatment in both strains (Fig. 2A, Table S6). Nearly 40% of them encode hypothetical or unknown proteins (Fig. 2B, Table S6); genes with an assigned name are included in Fig. 2A. For the genes with known function, the decrease in the expression of the *desB* gene, encoding an acyl-lipid desaturase, is most probably an acclimation response compensating for temperature-induced increase in membrane fluidity. Up-regulation of the *desB* gene in low temperatures and adjustment of lipid saturation are well known responses to low and high temperature [30], [31]. The heat shock genes have been shown to be rapidly but only transiently up-regulated upon heat shock [21]. Up-regulation of heat shock genes typically occurs within minutes and transcripts disappear during the first hours of heat treatment. Accordingly, none of the heat shock genes was up-regulated after a 24-h treatment at 40°C. The *hspA* gene was up-regulated in ΔrpoZ in standard conditions [10] but this difference between the strains disappeared after the mild heat treatment. The *groES* heat shock gene was down-regulated in both strains and in addition the *htpG* heat shock gene was down-regulated in CS (Table S2) and the *dnaJ* heat shock gene was down-regulated in ΔrpoZ (Table S4). In addition to heat shock proteins, some other proteins have been suggested to affect heat responses. The *clpB1* gene encoding a protease, and slr1674 (a hypothetical protein) have shown to affect rapid heat responses, whereas *hik34* (encoding a histidine kinase) and *hypA1* (encoding a hydrogenase formation protein) affect sustained thermotolerance of PSII, and *cpcC2* (encoding a phycobilisome rod linker polypeptide) is essential for both responses [32]. The *slr1674*, *hypA1* and *clpB1* genes were up-regulated in ΔrpoZ compared to CS at 40°C, whereas *cpcC2* was 1.5 fold down-regulated.

The vast majority of genes up-regulated upon a mild heat treatment in the control or ΔrpoZ strains belonged to functional categories hypothetical or unknown (Figs. 2B and 3, Table S6). The other large group of up-regulated genes was transport and binding proteins comprising 20 and 21 genes in CS and ΔrpoZ, respectively (Figs. 2B and 3). Many of them, including ammonium/methylammonium permeases, ABC-type basic amino acid and glutamine transporter, a permease protein for urea transporter and a manganese transporter (Table S6), were up-regulated in both strains. However, some transporters were up-regulated in one strain only, like nitrate/nitrite transporter genes, which were among the most highly up-regulated genes in ΔrpoZ, but were not up-regulated in CS. Some other differences in central nitrogen metabolism genes were detected in addition. The *nblA1* and *nblA2* genes encoding phycobilisome degradation proteins [33], [34] were up-regulated only in ΔrpoZ while *glnB*, encoding the nitrogen metabolism regulator protein PII [35], was up-regulated only in CS. Interestingly, Rre37, controlling some sugar catabolism genes in parallel with SigE mainly during nitrogen starvation [36], was up-regulated upon heat stress in both strains, but up-regulation of its target genes *glgP* and *glgX* was only detected in ΔrpoZ. Differential regulation of several genes involved in nitrogen metabolism may suggest that acclimation of nitrogen metabolism to elevated temperature fails to occur normally in ΔrpoZ.

Seven genes showed opposite expression change in ΔrpoZ and CS upon mild heat stress. Five genes were down-regulated in ΔrpoZ and up-regulated in CS, but only one of these genes, *trmD* encoding tRNA (guanine-N1-)-methyltransferase, has an assigned function (Fig. 2A). On the other hand, two genes were up-regulated in ΔrpoZ and down-regulated CS; these genes were *gcvP* encoding glycine dehydrogenase and *ccmR*, which encodes a repressor protein regulating many genes involved in carbon concentrating mechanisms [37]. In standard growth conditions, the *ccmR* gene is down-regulated simultaneously with the down-regulation of its target genes and operons [10] indicating complex regulation of carbon concentrating mechanisms in ΔrpoZ.

According to DNA microarray results, more than 80% that showed up or down regulation in ΔrpoZ did not respond similarly to a mild heat treatment in CS (Fig. 2A). Up-regulation of photosynthetic and respiratory genes was more common in ΔrpoZ than in CS (Figs. 2B and 3). Furthermore, many genes for biosynthesis of amino acids and cofactors, prosthetic groups and carriers were up-regulated upon heat stress in ΔrpoZ strain but only few in CS (Fig. 2B).

Although ΔrpoZ grew well in standard conditions, the DNA microarray analysis revealed that 187 genes were at least two-fold up-regulated and 212 genes down-regulated in ΔrpoZ cells compared to CS in standard growth conditions [10]. Our next question was whether the genes showing different response to mild-heat treatment in ΔrpoZ and CS were similarly or differently expressed in the standard conditions. The heat map reveals that numerous genes up-regulated upon heat stress in ΔrpoZ were actually down-regulated in ΔrpoZ compared to CS in standard conditions (Fig. 3). For example, genes encoding NADH dehydrogenase subunits that were shown to be down-regulated in ΔrpoZ in standard conditions [10] were up-regulated in ΔrpoZ upon heat treatment but not in CS (Fig. 3). Furthermore, many genes that were down-regulated upon mild heat stress in ΔrpoZ were found to be up-regulated in ΔrpoZ compared to CS in standard conditions (Fig. 3). In standard conditions we showed that recruitment of the primary σ factor SigA occurs less frequently in ΔrpoZ than in CS, which leads to down-regulation of many highly expressed genes in ΔrpoZ [10]. The physiological experiments using group 2 σ factor mutant strains have revealed that SigB and SigC factors are important for high temperature acclimation responses [14], [15], [25] and thus is tempting to speculate that the ω subunit not only affects the recruitment of SigA but also the recruitment of the other σ factors.

### RNA polymerase and total RNA contents decrease in mild heat stress more in CS than in ΔrpoZ

**Figure 4:**
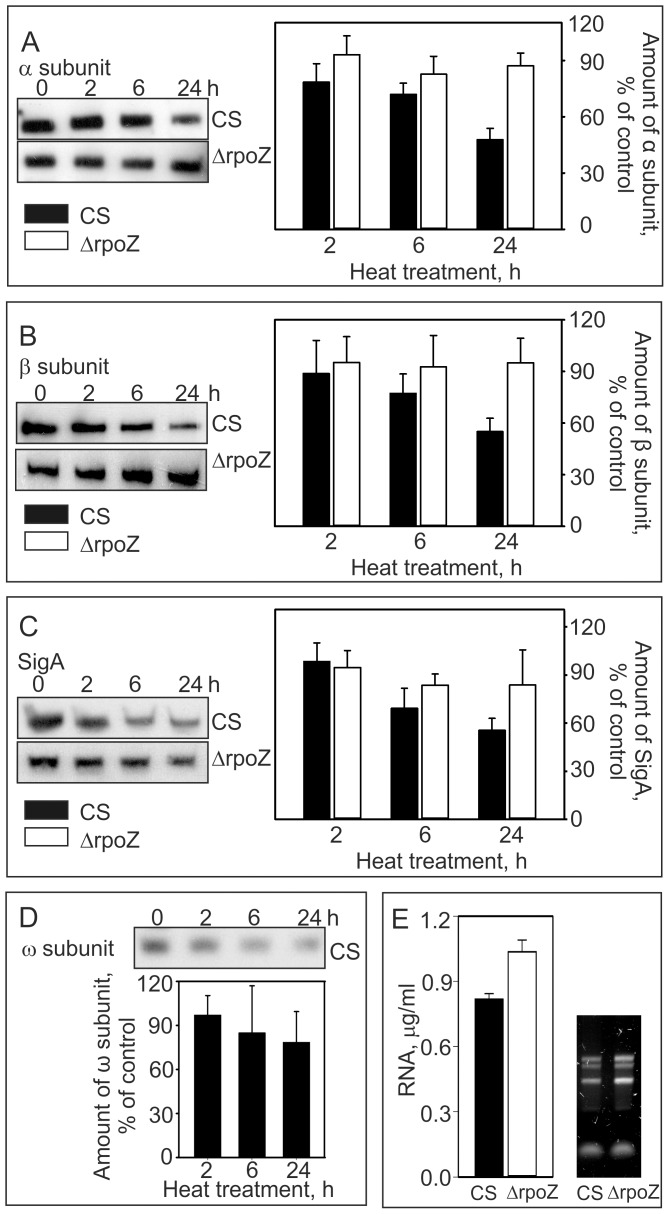
Contents of RNA polymerase and RNA in mild heat stress in the control and ΔrpoZ strains. Total proteins were isolated after 0, 2, 4 and 24 h treatments at 40°C, samples containing 50 µg of protein were separated with SDS-PAGE, and the amounts of the α (A), β (B), SigA (C) and ω (D) subunits of RNAP were determined by western blotting. (E) Total RNA content of cells after 24-h heat treatment. Total RNA content in 1-mL sample (OD_730_ = 1) of CS (black bars) and ΔrpoZ (white bars) cell cultures incubated for 24 h at 40°C. Each result represents the mean of three biological replicates and the error bars denote SEM. A 5-µl sample of isolated RNA was separated in 1.2% agarose gel and stained with ethidium bromide to visualize rRNA.

Next we analyzed the RNAP content of cells in mild heat stress. The cells were grown under standard conditions and then transferred to 40°C for 2, 6 or 24 h. Western blots showed a clear decrease of RNAP during the high temperature treatment in CS; after one day treatment, cells had lost 45% of the RNAP core subunits α and β (Fig. 4AB). On the contrary, the ΔrpoZ strain lost less than 10% of RNAP core subunits α and β (Fig. 4AB). In addition, the amount of the primary σ factor, SigA, decreased in heat stress; after 24-h heat treatment 45% and 17% of SigA was lost in CS and ΔrpoZ, respectively (Fig. 4C). The ω subunit decreased similarly in CS as the other RNAP core subunits (Fig. 4D). In accordance with decrease of RNAP in CS, the total RNA content of CS cells decreased from 1.2 µg/ml in cultures with OD_730_ = 1 [10] to 0.8 µg/ml after a 24-h treatment at 40°C (Fig. 4E). In the ΔrpoZ strain, the RNA content was similar as in CS in standard conditions [10]. The RNA content of ΔrpoZ decreased only 17% during the 24-h heat treatment (Fig. 4E) suggesting that the higher RNAP content of ΔrpoZ keeps transcription in ΔrpoZ more active than in CS in mild heat stress. However, the stability of transcripts is known to vary according to environmental cues [38] and we cannot rule out the possibility that the RNA contents of CS and ΔrpoZ are affected by RNA stability at high temperatures.

More than 90% of the total RNA in cells consists of rRNA, and analysis of total RNA by agarose gel electrophoresis revealed that the rRNA content of CS was lower than that of the ΔrpoZ strain (Fig. 4E). In *E.coli*, severe heat stress has shown to disturb ribosome assembly [39] and on the other hand, ribosomes form inactive 100S dimers when cells enter a non-growth mode in stationary phase [40], [41]. In our mild heat stress conditions, CS grew as well as in standard conditions, indicating that translation remained fully active although the rRNA content of the cells decreased. Increase in temperature speeds up enzyme reactions and a lower amount of ribosomes might provide fully active translation in a slightly elevated temperature. In the case of ΔrpoZ, further experiment are required to find out whether a high rRNA content directly affects ribosome content and whether all ribosomes are translationally active or not.

We used total RNA samples in DNA microarray analysis, and the decrease in the RNA content of the cells during mild heat stress might affect the DNA microarray results, as we do not know whether the mRNA/rRNA ratio remained similar in all samples. However, overall signal intensities in the DNA microarray raw data did not reveal any systematic differences between the treatments or the strains, suggesting that the mRNA/rRNA ratio was not drastically different between samples. The method used for data normalization was found to be important when time series samples were analyzed [42]; in pairwise comparisons, performed in the present study, the quantile method is regularly used.

### Photosynthetic capacity of ΔrpoZ decreased in mild heat stress

Photosynthesis is known to be a heat sensitive process. A 60-min heat treatment at 42°C was shown to reduce photosynthetic activity by 15% [43], and many parts of photosynthetic reactions, including carbon fixation by Rubisco and photosynthetic light reactions, especially the oxygen evolving complex of PSII, are known to be heat sensitive [44]. As many genes belonging to the category “photosynthesis and respiration”, showed differential response to heat in CS and ΔrpoZ (Figs. 2B and 3), we studied the acclimation of the photosynthetic machinery. To measure heat-induced changes, cells were grown in standard conditions and thereafter treated at 40°C for 24 h under constant illumination, PPFD 40 µmol m^−2^ s^−1^.

**Figure 5:**
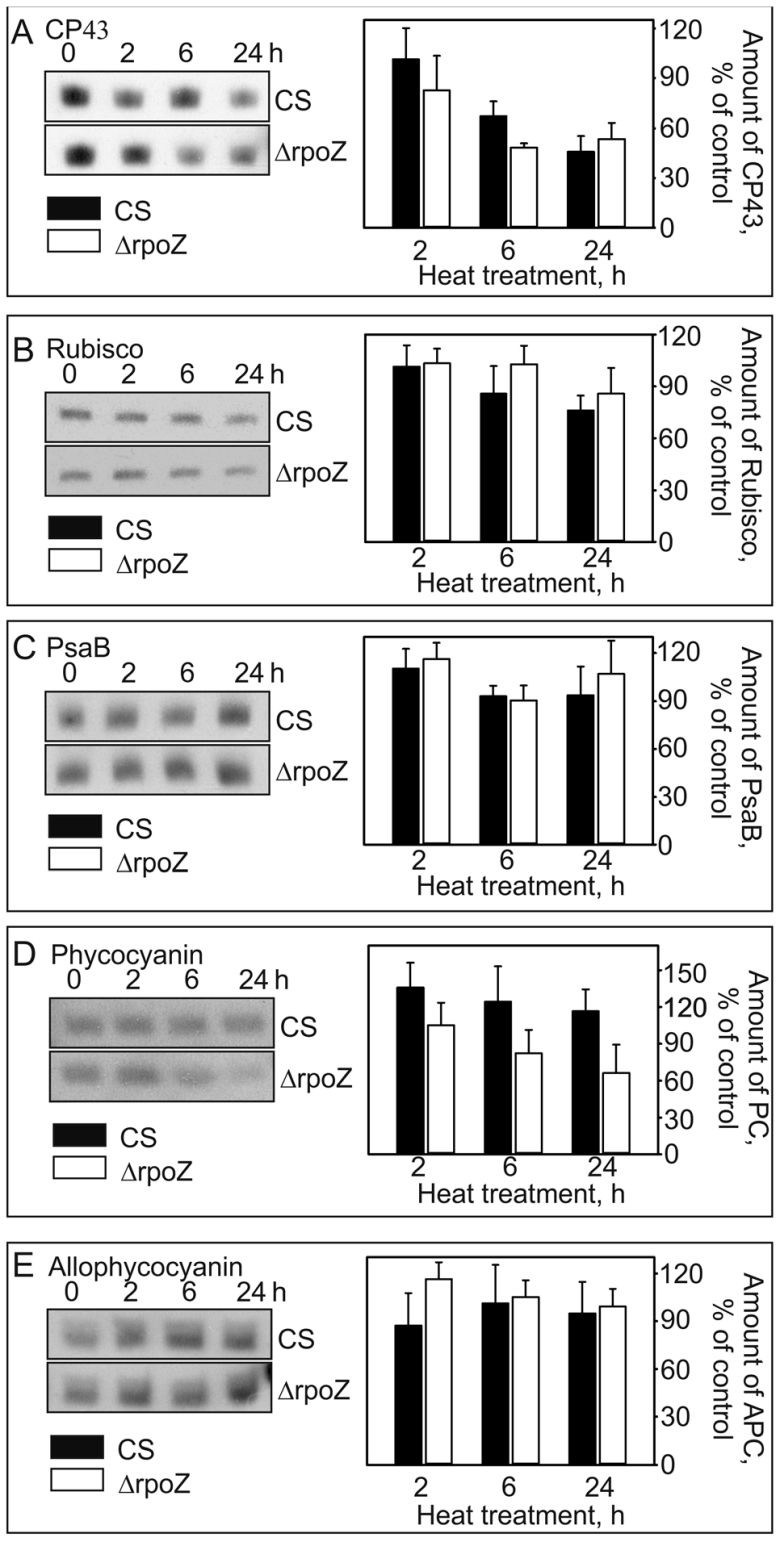
Changes in photosynthetic proteins during 24-h treatment at 40°C in CS and ΔrpoZ. Total proteins were isolated after 0, 2, 4 and 24 h treatments at 40°C, and solubilized proteins were separated with SDS-PAGE. The PSII core protein CP43 (A), the large RbcL subunit of Rubisco (B), PSI reaction center protein PsaB (C), and phycobilisome proteins phycocyanin (D) and allophycocyanin (E) were detected by western blotting with specific antibodies. Total proteins loaded were 5 µg in A and C, 10 µ in B and 1.6 µg in D and E. Each bar shows the mean of three biological replicates and the error bars denote SEM.

We detected the amounts of different photosynthetic complexes during the 24-h treatment at 40°C with western blotting. A clear decrease of PSII (measured using an antibody against the PSII core protein CP43) and 10 to 15% decrease of Rubisco (measured using an antibody against RbcL) occurred in both strains (Figs. 5A and 5B), while the PSI content (antibody against PSI reaction center protein PsaB) remained at the same level as in standard growth conditions (Fig. 5C). In CS, the phycobilisome antenna proteins phycocyanin and allophycocyanin remained constant during the 24-h heat treatment at 40°C (Figs. 5D and 5E). However, in ΔrpoZ the phycocyanin content of the cells decreased (Fig. 5D) although allophycocyanin (Fig. 5E) remained at the same level as in the control conditions. Interestingly, heat treatment induced up-regulation of the *nblA1* and *nblA2* genes (encoding the phycobilisome degradation proteins NblA1 and NblA2, respectively) in ΔrpoZ but not in CS (Fig. 3). NblA1 and NblA2 proteins form a heterodimer [33] that acts as an adaptor guiding the Clp protease to phycobilisomes [45]. These findings suggest that up-regulation of NblA proteins in ΔrpoZ upon heat stress induces degradation of phycobilisome rods that consist of phycocyanin.

**Figure 6:**
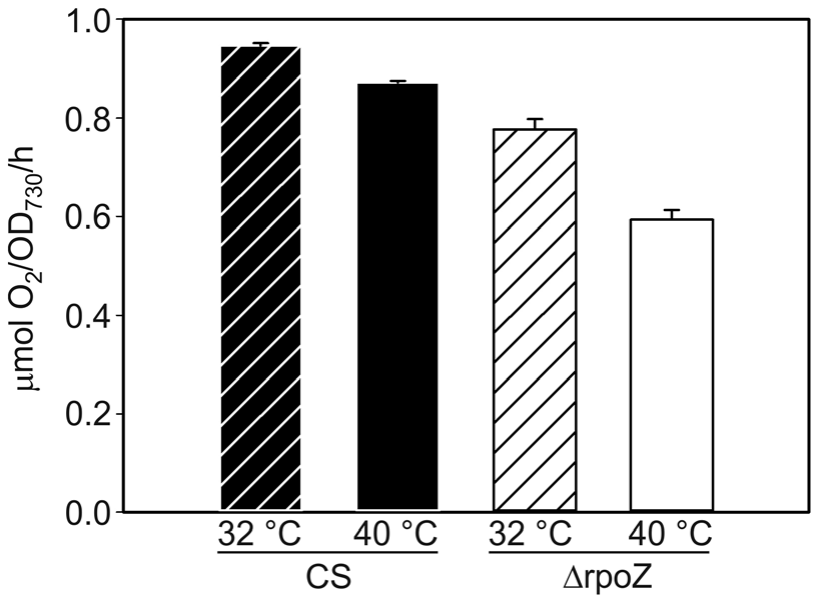
Acclimation of photosynthesis to mild heat stress in CS and ΔrpoZ. Light-saturated photosynthetic activity of CS and ΔrpoZ was measured in the standard growth conditions at 32°C and after 24-h of high temperature treatment at 40°C with a Clark type oxygen electrode. The results are means of three biological replicates and the error bars denote SEM.

After the 24-h treatment at 40°C, the light-saturated photosynthetic activity of CS, measured by oxygen evolution, was 92% of that measured in standard conditions (Fig. 6). In standard conditions, light-saturated photosynthetic activity of ΔrpoZ was circa 20% lower than in CS (Fig. 6A) and it further decreased in mild heat stress being only 68% of that measured in CS after 24-h treatment at 40°C (Fig. 6). The light-saturated PSII activities of the cells grown in mild heat stress, measured using a quinone electron acceptor, were 2.02±0.08 and 1.38±0.16 µmol O_2_/OD_730_/h in the control and ΔrpoZ strains, respectively, indicating that PSII of the ΔrpoZ strain was vulnerable to heat-treatment.

## Conclusions

**Figure 7:**
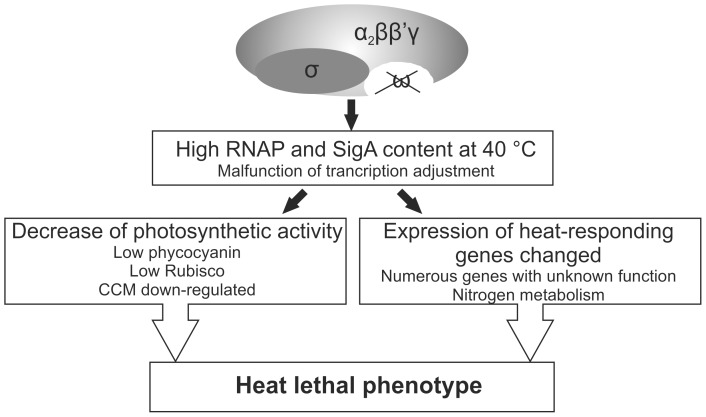
Cellular processes that respond differently to heat treatment in CS and ΔrpoZ.

The heat-lethal phenotype of ΔrpoZ strain emphasizes the view that the small ω subunit of RNAP is an important core polypeptide although cells can survive without it in optimal laboratory conditions. The total RNA content of the cells remains higher in ΔrpoZ than in CS during heat stress, and therefore the heat-lethal phenotype of ΔrpoZ is probably not caused by a decrease in active RNAP due to the proposed chaperone-like activity of the ω subunit. Instead, our data suggest that numerous heat acclimation processes malfunction in ΔrpoZ. As summarized in Fig. 7, these acclimation processes include adjustment of transcription, photosynthesis and nitrogen metabolism. Gene expression respond differently in ΔrpoZ and CS, and the data indicate that the small ω subunit affects expression of specific genes not only in standard growth conditions but also during heat stress.

## Materials and Methods

### Strains, growth conditions and growth measurements

The glucose tolerant control strain of *Synechocystis* sp. PCC 6803 [46], the ω subunit inactivation strain ΔrpoZ and the complementation strain ΔrpoZ+rpoZ [10] were grown in BG-11 medium supplemented with 20 mM Hepes pH 7.5. The OD_730_ of liquid cultures was set to 0.1 (0.35 µg of chlorophyll (Chl) *a*/ml), and the cells were grown (30 ml of cell culture in a 100-ml Erlenmeyer flask) at 32°C, 38°C or 40°C in air level CO_2_ under constant illumination at the PPFD of 40 µmol m^−2^ s^−1^ and shaking at 90 rpm. In some experiments, as indicated, BG-11 medium was supplemented with 20 mM Hepes, pH 8.3. Samples of dense cultures were diluted with BG-11 before the optical density was measured, so that OD_730_ did not exceed 0.4, and the dilutions were taken into account when the final results were calculated. All measurements were conducted on at least three independent biological replicates.

### Survival rates at 40°C

OD_730_ was set to 0.1, and cells were grown at 40°C for 24 h. The OD_730_ was measured, cells were diluted with fresh BG-11 medium to OD_730_ = 0.1. Then culture was serially diluted to 1∶10, 1∶100, 1∶1000 and 1∶10 000; and twenty drops containing 10 µl of the dilutions were spotted onto BG-11 plates. Plates were grown in standard conditions for one week, the colonies were counted and CFUs were calculated as CFUs/1-ml cell culture with OD_730_ = 0.1.

### DNA microarray analysis

For DNA microarray studies, OD_730_ was set to 0.1, and the cells were grown in standard growth conditions for three days. Then the samples from standard conditions (OD_730_ = 1, 40 ml) were harvested by centrifugation at 4500 g for 6 min at 4°C in pre-cooled centrifuge tubes [10] or cells were treated at 40°C under continuous illumination, PPFD 40 µmol m^−2^ s^−1^, for 24 h before harvest. The RNA was isolated using the hot-phenol method as described in [47], and further purified with RNeasy Mini Kit (Qiagen) to remove DNA contaminations. A 8×15 K custom *Synechocystis* sp. PCC 6803 array (Agilent) was used in microarray experiments [48], and hybridizations and data collection were done as described previously [49]. The data were normalized using the quantile method and the t-test was used to identify differentially expressed genes. A gene was considered differentially regulated if log_2_ of the fold change was ≥1 (at least two-fold up-regulated) or ≤−1 (down-regulated to one half or less) and P<0.05. Gene expression data were visualized with a heat map drawn with the open source software Multiple Experiment Viewer [50].

### Total RNA content of the cells

Cells were first grown in standard growth conditions and then treated at 40°C under continuous illumination, PPFD 40 µmol m^−2^ s^−1^, for 24 h before harvest. Total RNA was isolated with the hot-phenol method [47] from 1-ml of cell culture with OD_730_ = 1, and suspended in 12 µl of water. RNA concentration was measured with NanoDrop spectrophotometer and RNAS were visualized by running 5-µl samples on 1.2% agarose gels and staining the gels with ethidium bromide.

### Western blotting

Cells (25 ml; OD_730_ = 1; 3.5 µg Chl *a*/ml) were harvested from standard growth conditions, or treated at 40°C under continuous illumination (PPFD 40 µmol m^−2^ s^−1^) for 2, 6, or 24 h before harvesting. Total proteins were isolated as described previously [51]. Protein samples containing 1.6 µg (allophycocyanin, phycocyanin), 5 µg (CP43), 10 µg (PsaB, Rubisco), 20 µg (the ω subunit) or 50 µg (α, β, and SigA subunits of RNAP) of total proteins were solubilized for 10 min at 75°C and separated by 10% NEXT GEL SDS-PAGE (Amresco) according to the manufacturer's instructions. Proteins were transferred to Immobilon-P membrane (Millipore). Antibodies against allophycocyanin of *Porphyridium cruentum* (AS08 277), CP43 of *Arabidopsis thaliana* (AS11 1787), PsaB of *Arabidopsis thaliana* (AS10 695), phycocyanin of *Porphyridium cruentum* (AS08 278), and RbcL (AS03 037), and custom polyclonal antibodies recognizing α, β, ω and SigA subunits of *Synechocystis* RNAP [10] were purchased from Agrisera. The Goat Anti-Rabbit IgG (H+L) alkaline phosphatase conjugate (Zymed) and the CDP star chemiluminescence kit (New England Biolabs) were used for detection. Immunoblots were quantified with a FluorChem image analyzer (Alpha Innotech Corp.).

### Photosynthetic activity

Light-saturated photosynthetic activity *in vivo* was measured (1 ml sample, OD_730_ = 1) with a Clark type oxygen electrode (Hansatech Ltd.) at 32°C in BG-11 medium supplemented with 10 mM NaHCO_3_. The light-saturated PSII activity was measured using 0.7 mM 2,6-dichloro-*p*-benzoquinone as an artificial electron acceptor, and samples were also supplemented with 0.7 mM ferricyanide to keep the electron acceptor in oxidated form.

## Supporting Information

Table S1 **Genes at least two fold up-regulated in the control strain after a 24-h treatment at 40°C.**
(PDF)

Table S2 **Genes down-regulated to half or less in the control strain after a 24-h treatment at 40°C.**
(PDF)

Table S3 **Genes that were at least two fold up-regulated in ΔrpoZ after a 24-h treatment at 40°C.**
(PDF)

Table S4 **Genes down-regulated to half or less in the ΔrpoZ strain after a 24-h treatment at 40°C.**
(PDF)

Table S5 **List of genes included in** **Fig. 3** **and their expression data.**
(PDF)

Table S6 **List of genes that were similarly or oppositely regulated in CS and ΔrpoZ after heat treatment.**
(PDF)
