# Supplementary material for: The ω Subunit of RNA Polymerase Is Essential for Thermal Acclimation of the Cyanobacterium Synechocystis Sp. PCC 6803
Source: PLoS One. 2014 Nov 11;9(11):e112599. doi: 10.1371/journal.pone.0112599 (PMC4227741; doi:10.1371/journal.pone.0112599)
Supplement: Table S1 — Genes at least two fold up-regulated in the control strain after a 24-h treatment at 40°C. (PDF) [file pone.0112599.s001.pdf]

Table S1. Genes at least two fold up-regulated in the control strain after a 24-h treatment at 40 °C.

ORF	FC*	P value	Annotation/Function	Gene name	Functional category**
slr0288	2.65	0.042303	glutamate--ammonia ligase	*glnN*	A
slr1756	2.01	0.012725	glutamate--ammonia ligase	*glnA*	A
slr1898	1.27	0.031619	N-acetylglutamate kinase	*argB*	A
ssl0707	1.42	0.030626	nitrogen regulatory protein P-II	*glnB*	A
sll1514	1.45	0.039969	16.6 kDa small heat shock protein, molecular chaperone	*hspA,*	D
sll1694	1.88	0.000174	pilin polypeptide PilA1	*pilA1*	D
sll1695	2.19	0.000052	pilin polypeptide PilA2	*pilA2*	D
slr1289	1.81	0.009670	isocitrate dehydrogenase (NADP+)	*icdA*	F
ssl2559	1.23	0.002315	ferredoxin		H
sll0248	1.07	0.047051	flavodoxin	*isiB*	H
sll0247	1.86	0.012778	iron-stress chlorophyll-binding protein	*isiA*	H
ssr0390	1.04	0.044694	photosystem I reaction center subunit X	*psaK1*	H
slr0851	1.20	0.035004	type 2 NADH dehydrogenase	*ndbA*	H
sll1161	1.71	0.000343	probable adenylate cyclase	*cya3*	J
sll0782	1.94	0.020364	transcriptional regulator		J
slr1759	1.07	0.037249	two-component hybrid sensor and regulator	*hik14*	J
sll1330	1.83	0.019695	two-component system response regulator	*rre37*	J
slr1543	1.00	0.012326	DNA-damage-inducible protein F		K2
slr1912	1.67	0.002181	putative PP2C-type protein phosphatase		L
sll2008	1.08	0.015432	processing protease	*prp1*	M
sll1198	1.73	0.004000	tRNA (guanine-N1)-methyltransferase	*trmD*	M
slr1316	1.15	0.008354	ABC-type iron(III) dicitrate transport system permease protein	*fecC*	N
sll0108	2.23	0.021606	ammonium/methylammonium permease	*amt1*	N
sll0537	1.04	0.006726	ammonium/methylammonium permease	*amt3*	N
sll1017	2.17	0.036233	ammonium/methylammonium permease	*amt2*	N
slr1735	1.13	0.036765	ATP-binding subunit of the ABC-type Bgt permease for basic amino acids and glutamine	*bgtA*	N
sll1404	2.10	0.004326	biopolymer transport ExbB protein homolog	*exbB3*	N
sll1405	1.97	0.004938	biopolymer transport ExbD protein homolog	*exbD*	N
sll1406	1.69	0.001927	ferrichrome-iron receptor	*fhuA*	N
slr1295	2.19	0.000083	iron transport system substrate-binding protein	*futA1*	N
slr0513	1.11	0.000820	iron transport system substrate-binding protein, periplasmic protein	*futA2*	N
slr1318	1.11	0.001189	iron(III) dicitrate transport system ATP-binding protein	*fecE*	N
slr0096	1.07	0.018806	low affinity sulfate transporter		N
sll1599	1.63	0.015019	manganese transport system ATP-binding protein MntA	*mntA*	N
sll1598	1.88	0.011558	Mn transporter MntC	*mntC*	N
slr1488	1.25	0.000606	multidrug resistance family ABC transporter		N
slr0944	1.03	0.028080	multidrug-efflux transporter	*arsB*	N
sll1270	1.95	0.015768	periplasmic substrate-binding and integral membrane protein of the ABC-type Bgt permease for basic amino acids and glutamine BgtB	*bgtB*	N
sll0536	2.09	0.015045	probable potassium channel protein	*kchX*	N
sll0764	1.34	0.005542	urea transport system ATP-binding protein	*urtD*	N
slr1200	3.12	0.005600	urea transport system permease protein	*urtB*	N
slr1201	1.87	0.013713	urea transport system permease protein	*urtC*	N
slr2002	1.66	0.036906	cyanophycin synthetase	*cphA*	O
sll1159	2.86	0.000003	probable bacterioferritin comigratory protein		O

ORF	FC*	P value	Annotation/Function	Gene name	Functional category**
sll1407	1.30	0.003801	probable methyltransferase		O
sll0335	**1.62**	**0.022188**	hypothetical protein		P
sll0944	1.25	0.027989	hypothetical protein		P
sll1119	2.65	0.014692	hypothetical protein		P
sll1158	3.42	0.000001	hypothetical protein		P
sll1160	2.07	0.000036	hypothetical protein		P
sll1469	1.02	0.001049	hypothetical protein		P
sll1696	1.41	0.000093	hypothetical protein		P
slr0144	1.14	0.000628	hypothetical protein		P
slr0146	1.03	0.001516	hypothetical protein		P
slr1152	2.08	0.001139	hypothetical protein	rfrK	P
slr1290	1.05	0.023606	hypothetical protein		P
slr1770	1.16	0.037408	hypothetical protein		P
slr1913	1.01	0.002005	hypothetical protein		P
ssl0331	1.12	0.035017	hypothetical protein		P
ssl1762	1.21	0.043332	hypothetical protein		P
sll0327	1.13	0.048209	unknown protein		Z
sll0441	1.35	0.011751	unknown protein		Z
sll0733	2.06	0.011220	unknown protein		Z
sll0783	3.84	0.027555	unknown protein		Z
slr0145	1.14	0.000459	unknown protein		Z
slr0442	1.29	0.005213	unknown protein		Z
slr1484	1.80	0.001646	unknown protein		Z
slr1681	1.17	0.020969	unknown protein		Z
slr1726	1.06	0.001357	unknown protein		Z
slr1920	1.10	0.026555	unknown protein		Z
ssl2384	2.15	0.000229	unknown protein		Z

*FC: $\log_2$ of fold change.

**The categories are listed according to Cyanobase (see Fig. 2).
