# Supplementary material for: The ω Subunit of RNA Polymerase Is Essential for Thermal Acclimation of the Cyanobacterium Synechocystis Sp. PCC 6803
Source: PLoS One. 2014 Nov 11;9(11):e112599. doi: 10.1371/journal.pone.0112599 (PMC4227741; doi:10.1371/journal.pone.0112599)
Supplement: Table S2 — Genes down-regulated to half or less in the control strain after a 24-h treatment at 40°C. (PDF) [file pone.0112599.s002.pdf]

Table S2. Genes down-regulated to half or less in the control strain after a 24-h treatment at 40 °C.

ORF	FC*	P value	Function	Gene name (if assigned)	Functional category**
sll0450	-2.386	0.0002	cytochrome b subunit of nitric oxide reductase	*norB*	A
sll1688	-1.017	0.0029	threonine synthase	*thrC*	A
slr0426	-1.018	0.0405	GTP cyclohydrolase I	*folE*	B
slr0749	-1.108	0.0042	light-independent protochlorophyllide reductase iron protein subunit ChlL	*chlL*	B
slr1434	-1.1	0.0068	pyridine nucleotide transhydrogenase beta subunit	*pntB*	B
slr0984	-1.371	0.0001	CDP-glucose 4,6-dehydratase	*rfbG*	C
slr1072	-1.108	0.0001	GDP-D-mannose dehydratase	*rfbD, yefA*	C
sll1724	-1.094	0.0243	probable glycosyltransferase	*icsA*	C
slr1064	-1.662	0.0008	probable glycosyltransferase	*rfbU, mtfA*	C
slr1351	-1.013	0.0415	UDP-N-acetylmuramoylalanyl-D-glutamyl-2 6-diaminopimelate--D-alanyl-D-alanine ligase	*murF*	C
slr2075	-1.083	0.0106	10kD chaperonin	*groES*	D
sll0430	-1.732	0.0050	HtpG, heat shock protein 90, molecular chaperone	*htpG*	D
slr0427	-1.001	0.0023	putative competence-damage protein	*psbA2*	D
ssl2922	-1.153	0.0251	similar to virulence-associated protein VapB	*vapB*	D
sml0009	-1.138	0.0227	similar to virulence-associated protein VapC		D
slr1705	-1.256	0.0038	aspartoacylase	*aspA*	F
slr0293	-1.174	0.0007	glycine dehydrogenase	*gcvP*	F
slr1350	-1.145	0.0005	acyl-lipid desaturase (delta 12)	*desA*	G
sll1441	-2.398	0.0019	acyl-lipid desaturase (omega-3)	*desB*	G
sll0330	-1.192	0.0470	sepiapterine reductase	*fabG*	G
slr1291	-1.868	0.0002	NADH dehydrogenase subunit 4	*ndhD2*	H
slr2007	-3.324	0.0016	NADH dehydrogenase subunit 4	*ndhD5*	H
slr2009	-1.738	0.0023	NADH dehydrogenase subunit 4	*ndhD6*	H
ssl3044	-1.233	0.0069	probable ferredoxin		H
slr1164	-1.405	0.0000	ribonucleotide reductase subunit alpha	*nrdA, dnaF*	I
sll1626	-1.328	0.0082	LexA repressor	*lexA*	J
sll1594	-3.105	0.0003	ndhF3 operon transcriptional regulator, LysR family protein	*ccmR, ndhR*	J
slr1214	-1.087	0.0143	two-component response regulator	*rre15*	J
slr1285	-1.056	0.0159	two-component sensor histidine kinase	*hik34*	J
sll1772	-1.313	0.0236	DNA mismatch repair protein MutS	*mutS*	K2
slr0790	-1.473	0.0440	similar to ultraviolet light resistance protein B	*umuC*	K2
slr0653	-1.118	0.0001	principal RNA polymerase sigma factor SigA	*sigA*	L
sll0555	-1.258	0.0256	methionine aminopeptidase	*map-3, mapC*	M
slr1592	-2.288	0.0002	probable pseudouridine synthase		M
sll1263	-1.364	0.0028	cation efflux system protein		N
slr0067	-1.233	0.0001	MRP protein homolog		O
slr1410	-1.04	0.0080	periplasmic WD-repeat protein		O
slr1019	-1.491	0.0454	phenazine biosynthetic protein PhzF homolog		O
slr1063	-1.573	0.0016	probable glycosyltransferase		O
slr1065	-1.493	0.0003	probable glycosyltransferase		O
slr1076	-1.291	0.0000	probable glycosyltransferase		O
slr1077	-1.195	0.0000	probable glycosyltransferase	*gumH*	O
slr1610	-1.118	0.0297	putative C-3 methyl transferase		O
sll0222	-1.04	0.0155	putative purple acid phosphatase	*phoA*	O
sll0185	-1.601	0.0342	hypothetical protein		P

ORF	FC*	P value	Function	Gene name (if assigned)	Functional category**
sll0360	-1.004	0.0148	hypothetical protein		P
sll0451	-1.148	0.0142	hypothetical protein		P
sll0529	-1.174	0.0295	hypothetical protein		P
sll0822	-1.056	0.0122	hypothetical protein		P
sll1504	-1.574	0.0014	hypothetical protein		P
sll1505	-1.938	0.0145	hypothetical protein		P
sll1516	-1.18	0.0049	hypothetical protein		P
sll1911	-1.144	0.0218	hypothetical protein		P
slr0870	-1.072	0.0008	hypothetical protein		P
slr0959	-1.676	0.0009	hypothetical protein		P
slr1069	-1.628	0.0003	hypothetical protein		P
slr1472	-1.06	0.0075	hypothetical protein		P
slr1677	-1.073	0.0371	hypothetical protein		P
slr1692	-1.06	0.0294	hypothetical protein		P
slr2006	-3.627	0.0012	hypothetical protein	*mrpC*	P
slr2008	-1.898	0.0004	hypothetical protein	*mrpC*	P
slr2010	-1.963	0.0016	hypothetical protein	*mrpE*	P
slr2011	-1.734	0.0020	hypothetical protein	*mrpA*	P
slr2012	-1.581	0.0010	hypothetical protein	*mrpB*	P
slr2013	-1.248	0.0007	hypothetical protein		P
ssl2874	-1.233	0.0011	hypothetical protein		P
ssl2920	-1.532	0.0232	hypothetical protein		P
ssl2921	-1.398	0.0310	hypothetical protein		P
ssr1251	-1.428	0.0173	hypothetical protein		P
ssr2062	-1.269	0.0038	hypothetical protein		P
ssr2998	-1.016	0.0077	hypothetical protein		P
ssr3409	-1.583	0.0008	hypothetical protein	*mrpF*	P
ssr3410	-1.637	0.0021	hypothetical protein	*mrpG*	P
sll1483	-1.04	0.0031	periplasmic protein, similar to transforming growth factor induced protein		P
slr0708	-1.098	0.0102	periplasmic protein		Z
sll0263	-1.543	0.0052	unknown protein		Z
sll0710	-1.299	0.0004	unknown protein		Z
sll1239	-1.395	0.0371	unknown protein		Z
sll1241	-1.677	0.0099	unknown protein		Z
sll1611	-1.11	0.0099	unknown protein		Z
slr0262	-1.033	0.0208	unknown protein		Z
slr0871	-1.467	0.0091	unknown protein		Z
slr1066	-1.328	0.0038	unknown protein		Z
slr1071	-1.59	0.0001	unknown protein		Z
slr1073	-1.404	0.0000	unknown protein		Z
slr1074	-1.575	0.0004	unknown protein		Z
slr1383	-1.178	0.0251	unknown protein		Z
slr1618	-1.058	0.0027	unknown protein		Z
ssr2153	-1.416	0.0040	unknown protein		Z
ssr2194	-1.923	0.0011	unknown protein		Z

*FC: $\log_2$ of fold change.

**The categories are listed according to Cyanobase (see Fig. 2).
