# Supplementary material for: The ω Subunit of RNA Polymerase Is Essential for Thermal Acclimation of the Cyanobacterium Synechocystis Sp. PCC 6803
Source: PLoS One. 2014 Nov 11;9(11):e112599. doi: 10.1371/journal.pone.0112599 (PMC4227741; doi:10.1371/journal.pone.0112599)
Supplement: Table S3 — Genes that were at least two fold up-regulated in ΔrpoZ after a 24-h treatment at 40°C. (PDF) [file pone.0112599.s003.pdf]

Table S3. Genes that were at least two fold up-regulated in ΔrpoZ after a 24-h treatment at 40 °C.

ORF	FC*	P value	Function	Gene name (if assigned)	Functional category**
sll0784	2.42	0.000652	nitrilase	*merR*	A
sll1454	3.99	0.000108	ferredoxin-nitrate reductase	*narB*	A
sll1499	1.46	0.005635	ferredoxin-dependent glutamate synthase	*glsF, gltS*	A
sll1883	1.08	0.001408	arginine biosynthesis bifunctional protein ArgJ	*argJ*	A
slr0077	1.15	0.011710	cysteine desulfurase	*sufS, nifS*	A
slr0186	1.09	0.001845	2-isopropylmalate synthase	*leuA*	A
slr0288	1.79	0.000383	glutamate--ammonia ligase	*glnN*	A
slr0898	3.91	0.000019	ferredoxin--nitrite reductase	*nirA*	A
slr0899	3.91	0.000675	cyanate lyase	*cynS*	A
slr0963	1.39	0.002702	ferredoxin-sulfite reductase	*sir*	A
slr1756	1.79	0.000015	glutamate--ammonia ligase	*glnA*	A
slr0839	1.12	0.000000	ferrochelatase	*hemH, scpA*	B
slr0900	3.35	0.000077	molybdopterin biosynthesis MoeA protein	*moeA*	B
slr0901	3.36	0.000311	molybdopterin biosynthesis protein A	*moaA*	B
slr0902	2.10	0.000631	molybdenum cofactor biosynthesis protein C	*moaC*	B
slr1171	1.01	0.014274	glutathione peroxidase-like NADPH peroxidase,glutathione peroxidase	*gpx1*	B
slr1238	1.02	0.025763	glutathione synthetase	*gshB*	B
slr1254	1.74	0.008186	phytoene dehydrogenase (phytoene desaturase)	*crtP,*	B
slr1300	1.15	0.000016	similar to 2-octaprenyl-6-methoxyphenol hydroxylase	*ubiH, visB*	B
ssr1527	2.53	0.001561	probable molybdopterin [MPT] converting factor, subunit 1	*moaD*	B
ssr2061	1.38	0.000945	glutaredoxin	*grxA,, grxC*	B
sll1271	1.64	0.010011	probable porin; major outer membrane protein		C
slr0827	1.10	0.000153	alanine racemase	*dal, alr*	C
slr0993	1.38	0.003652	putative peptidase	*nlpD*	C
sll1294	2.48	0.000320	methyl-accepting chemotaxis protein	*taxD2*	D
sll1666	1.51	0.001748	DnaJ-like protein	*dnaJ*	D
slr0228	1.04	0.000713	cell division protein FtsH	*ftsH2*	D
slr1604	1.34	0.014578	cell division protein FtsH	*ftsH3*	D
sll0220	1.12	0.000638	L-glutamine:D-fructose-6-P amidotransferase	*glmS*	E
sll1676	1.38	0.000310	4-alpha-glucanotransferase	*malQ*	E
slr1367	1.68	0.003586	glycogen phosphorylase	*glgP2*	E
slr1857	2.32	0.000308	Isoamylase	*glgX*	E
sll0018	1.57	0.000002	fructose-bisphosphate aldolase, class II	*fbaA, fda*	F
sll0404	1.26	0.001148	glycolate oxidase subunit GlcD	*glcD*	F
sll1077	1.34	0.003890	agmatinase	*speB2*	F
sll1234	1.47	0.002990	adenosylhomocysteinase	*ahcY*	F
sll1498	1.62	0.000087	carbamoyl-phosphate synthase small chain	*carA, pyrA*	F
slr0293	1.71	0.002174	glycine dehydrogenase	*gcvP*	F
slr1289	1.77	0.000197	isocitrate dehydrogenase (NADP+)	*icd*	F
slr0574	1.00	0.000280	cytochrome P450	*cyp*	G
sll0519	1.30	0.003684	NADH dehydrogenase subunit 1	*ndhA*	H
sll0520	1.21	0.004315	NADH dehydrogenase subunit NdhI	*ndhI*	H
sll1031	1.09	0.047029	CO_2 concentrating mechanism protein CcmM, putative carboxysome structural protein	*ccmM*	H

ORF	FC*	P value	Function	Gene name (if assigned)	Functional category**
sll1732	1.92	0.001696	NADH dehydrogenase subunit 5 (involved in low CO2-inducible, high affinity CO2 uptake)	*ndhF3*	H
sll1733	1.36	0.031134	NADH dehydrogenase subunit 4 (involved in low CO2-inducible, high affinity CO2 uptake)	*ndhD3*	H
slr0851	1.31	0.000700	type 2 NADH dehydrogenase	*ndbA*	H
slr1279	1.56	0.003995	NADH dehydrogenase subunit 3	*ndhC*	H
slr1280	1.47	0.004267	NADH dehydrogenase subunit NdhK	*ndhK*	H
slr1281	1.70	0.000902	NADH dehydrogenase subunit I	*ndhJ*	H
slr1379	1.11	0.003758	quinol oxidase subunit I	*cydA*	H
slr1380	1.30	0.014901	quinol oxidase subunit II	*cydB*	H
slr1643	1.11	0.000272	ferredoxin-NADP oxidoreductase	*petH*	H
slr2059	1.28	0.009583	iron-sulfur cluster binding protein homolog		H
ssl0452	2.54	0.000079	phycobilisome degradation protein NblA	*nblA1*	H
ssl0453	2.38	0.000081	phycobilisome degradation protein NblA	*nblA2*	H
ssl2559	2.26	0.004072	ferredoxin		H
sll0567	1.24	0.000174	ferric uptake regulation protein	*fur*	J
sll0782	2.00	0.011603	transcriptional regulator		J
sll1161	4.55	0.000107	probable adenylate cyclase	*cya3*	J
sll1291	1.70	0.000275	two-component response regulator	*rre12*	J
sll1292	1.09	0.000590	two-component response regulator	*rre11*	J
sll1296	2.12	0.000281	two-component hybrid sensor and regulator	*hik39*	J
sll1330	1.40	0.012189	two-component system response regulator	*rre37*	J
sll1594	1.53	0.038007	ndhF3 operon transcriptional regulator, LysR family protein	*ccmR, ndhR,*	J
sll1670	1.04	0.004633	heat-inducible transcription repressor HrcA homolog	*hrcA*	J
slr1594	2.54	0.000965	two-component response regulator PatA subfamily	*rre5*	J
slr1860	1.54	0.003527	carbon metabolisms regulatory protein IcfG	*icfG*	J
slr1322	1.42	0.000324	putative modulator of DNA gyrase; TldD		K2
sll1787	1.34	0.008475	RNA polymerase beta subunit	*rpoB*	L
sll2012	1.30	0.002599	group2 RNA polymerase sigma factor SigD	*sigD*	L
slr1856	1.72	0.002684	phosphoprotein substrate of icfG gene cluster	*icfG*	L
slr1859	1.91	0.008937	anti-sigma f factor antagonist		L
slr1861	1.20	0.002641	probable sigma regulatory factor		L
sll2008	1.78	0.000246	processing protease	*prp1*	M
sll2009	1.84	0.000339	processing protease	*prp2*	M
slr0008	1.02	0.002629	carboxyl-terminal processing protease	*ctpA*	M
slr1204	1.00	0.013016	protease	*degP*	M
sll0108	2.08	0.000469	ammonium/methylammonium permease	*amt1*	N
sll0536	1.36	0.008087	probable potassium channel protein	*kchX*	N
sll0834	1.28	0.003290	low affinity sulfate transporter	*bicA*	N
sll1017	1.30	0.000222	ammonium/methylammonium permease	*amt2*	N
sll1081	2.13	0.001647	ABC transport system permease protein		N
sll1082	1.25	0.000296	ABC transport system ATP-binding protein		N
sll1180	1.09	0.019978	toxin secretion ABC transporter ATP-binding protein	*hlyB*	N
sll1270	1.98	0.012117	periplasmic substrate-binding and integral membrane protein of the ABC-type Bgt permease for basic amino acids and glutamine BgtB	*bgtB*	N
sll1450	4.37	0.000003	nitrate/nitrite transport system substrate-binding protein	*nrtA*	N
sll1451	4.70	0.000135	nitrate/nitrite transport system permease protein	*nrtB*	N

ORF	FC*	P value	Function	Gene name (if assigned)	Functional category**
sll1452	4.30	0.000235	nitrate/nitrite transport system ATP-binding protein	*nrtC*	N
sll1453	4.50	0.000062	nitrate/nitrite transport system ATP-binding protein	*nrtD*	N
sll1481	1.02	0.001119	ABC-transporter membrane fusion protein		N
sll1482	1.37	0.001248	ABC transporter permease protein		N
sll1598	1.70	0.000111	Mn transporter MntC	*mntC*	N
sll1599	1.47	0.000194	manganese transport system ATP-binding protein MntA	*mntA*	N
slr0447	1.30	0.002035	periplasmic ABC-type urea transport system substrate-binding protein	*urtA*	N
slr0944	1.91	0.000428	multidrug-efflux transporter	*arsB*	N
slr1200	1.51	0.000453	urea transport system permease protein	*urtB*	N
slr1735	1.39	0.000727	ATP-binding subunit of the ABC-type Bgt permease for basic amino acids and glutamine	*bgtA*	N
sll0217	1.20	0.000966	flavoprotein	*flv4*	O
sll0550	1.18	0.018284	flavoprotein	*flv3*	O
sll1078	1.57	0.006929	putative hydrogenase expression/formation protein HypA	*hypA2*	O
sll1079	1.57	0.000616	putative hydrogenase expression/formation protein HypB	*hypB*	O
sll1154	1.47	0.000124	putative antibiotic efflux protein	*norA*	O
sll1159	6.08	0.000030	probable bacterioferritin comigratory protein		O
sll1297	1.55	0.009001	probable dioxygenase, Rieske iron-sulfur component	*pobA*	O
sll1305	1.71	0.001640	probable hydrolase		O
sll1621	1.01	0.030354	AhpC/TSA family protein	*type II prx*	O
slr0541	1.01	0.010265	probable amidotransferase		O
slr0626	1.04	0.000335	probable glycosyltransferase		O
slr0665	1.19	0.001069	aconitate hydratase	*acnB*	O
slr0756	1.48	0.010125	circadian clock protein KaiA homolog	*kaiA*	O
slr0945	1.24	0.010417	arsenical resistance protein ArsH homolog	*arsH*	O
slr0946	1.47	0.005040	arsenate reductase	*arsC*	O
slr1498	1.05	0.000067	putative hydrogenase expression/formation protein HypD	*hypD*	O
slr1675	1.02	0.019546	putative hydrogenase expression/formation protein HypA1	*hypA1*	O
slr1853	2.09	0.000225	carboxymuconolactone decarboxylase		O
slr2002	1.65	0.004526	cyanophycin synthetase	*cphA*	O
sll0036	1.01	0.044721	hypothetical protein		P
sll0218	1.15	0.008954	hypothetical protein		P
sll0261	1.36	0.000620	hypothetical protein		P
sll0498	1.39	0.000053	hypothetical protein		P
sll0549	1.64	0.000006	hypothetical protein		P
sll0787	2.85	0.000353	hypothetical protein		P
sll0812	1.05	0.001200	hypothetical protein		P
sll0837	1.27	0.040676	periplasmic protein, function unknown		P
sll0846	1.41	0.010167	hypothetical protein		P
sll0888	1.26	0.002294	hypothetical protein		P
sll0944	1.10	0.037213	hypothetical protein		P
sll0983	1.11	0.007552	hypothetical protein		P
sll1039	1.24	0.002111	hypothetical protein		P
sll1080	1.39	0.000801	ABC transport system substrate-binding protein		P
sll1119	2.21	0.001101	hypothetical protein		P

ORF	FC*	P value	Function	Gene name (if assigned)	Functional category**
sll1158	7.90	0.000003	hypothetical protein		P
sll1160	4.17	0.000134	hypothetical protein		P
sll1162	1.36	0.000843	hypothetical protein		P
sll1251	1.84	0.000357	hypothetical protein		P
sll1314	1.21	0.001952	putative C4-dicarboxylase binding protein, periplasmic protein	*dctP*	P
sll1455	1.01	0.026089	hypothetical protein	*narM*	P
sll1541	1.16	0.021036	hypothetical protein	*syc2*	P
sll1734	1.17	0.009812	protein involved in low CO2-inducible, high affinity CO2 uptake	*cupA*	P
sll1783	1.47	0.000251	hypothetical protein		P
slr0076	1.14	0.025931	hypothetical protein	*sufD, sufB*	P
slr0292	2.48	0.000157	hypothetical protein		P
slr0300	1.61	0.001692	hypothetical protein		P
slr0320	1.14	0.000380	hypothetical protein		P
slr0397	1.12	0.041719	hypothetical protein		P
slr0751	1.25	0.001290	hypothetical protein		P
slr0769	1.04	0.001731	hypothetical protein		P
slr0964	1.23	0.028925	hypothetical protein		P
slr1119	1.55	0.000542	hypothetical protein		P
slr1220	1.95	0.016569	hypothetical protein		P
slr1276	1.23	0.002829	hypothetical protein	*pilO*	P
slr1290	1.01	0.001034	hypothetical protein		P
slr1376	1.19	0.015009	hypothetical protein		P
slr1593	1.50	0.000070	hypothetical protein	*ylmD*	P
slr1611	1.30	0.001130	hypothetical protein		P
slr1612	1.54	0.010136	hypothetical protein		P
slr1623	1.02	0.013918	hypothetical protein	*ndhM*	P
slr1660	1.09	0.008884	hypothetical protein		P
slr1674	1.40	0.000378	hypothetical protein		P
slr1717	1.06	0.000039	hypothetical protein		P
slr1753	1.04	0.009815	hypothetical protein		P
slr1770	2.34	0.000005	hypothetical protein		P
slr1799	1.13	0.000869	hypothetical protein		P
slr1906	1.68	0.002404	hypothetical protein		P
slr2125	1.09	0.006743	hypothetical protein		P
ssl0352	1.07	0.004197	hypothetical protein		P
ssl1762	1.57	0.003395	hypothetical protein		P
ssr0550	1.28	0.009572	hypothetical protein		P
sll0172	1.07	0.005822	periplasmic protein, function unknown		Z
sll0188	1.39	0.001958	unknown protein		Z
sll0225	1.20	0.002315	unknown protein		Z
sll0293	1.63	0.000084	unknown protein		Z
sll0733	2.06	0.009725	unknown protein		Z
sll0783	4.55	0.000070	unknown protein		Z
sll0785	2.92	0.002078	unknown protein		Z
sll0786	3.01	0.000527	unknown protein		Z

ORF	FC*	P value	Function	Gene name (if assigned)	Functional category**
sll0982	1.08	0.005930	unknown protein		Z
sll1163	1.49	0.001232	unknown protein		Z
sll1293	1.29	0.012304	unknown protein	*taxW2*	Z
sll1304	1.64	0.000326	unknown protein		Z
sll1306	1.11	0.001007	periplasmic protein, function unknown		Z
sll1396	1.40	0.000685	unknown protein		Z
sll1784	1.86	0.000050	periplasmic protein, function unknown		Z
sll1785	1.06	0.001021	periplasmic protein, function unknown	*cucA*	Z
slr0168	1.18	0.004442	unknown protein		Z
slr0273	1.10	0.008904	unknown protein		Z
slr0345	1.03	0.000196	unknown protein		Z
slr0617	1.08	0.036572	unknown protein		Z
slr1135	1.24	0.011318	unknown protein		Z
slr1852	2.46	0.000276	unknown protein		Z
slr1854	1.96	0.001853	unknown protein		Z
slr1855	1.95	0.000338	unknown protein		Z
slr2018	1.11	0.003064	unknown protein		Z
ssl1464	1.16	0.017312	unknown protein		Z
ssl1493	1.01	0.000184	unknown protein		Z
ssl2501	1.49	0.002722	unknown protein		Z
ssl2502	1.42	0.003643	unknown protein		Z
ssl2507	1.77	0.001788	unknown protein		Z
ssl2814	4.86	0.000019	unknown protein		Z
ssl3410	2.01	0.000015	unknown protein		Z

*FC: $\log_2$ of fold change.

**The categories are listed according to Cyanobase (see Fig. 2).
