# Supplementary material for: The ω Subunit of RNA Polymerase Is Essential for Thermal Acclimation of the Cyanobacterium Synechocystis Sp. PCC 6803
Source: PLoS One. 2014 Nov 11;9(11):e112599. doi: 10.1371/journal.pone.0112599 (PMC4227741; doi:10.1371/journal.pone.0112599)
Supplement: Table S4 — Genes down-regulated to half or less in the ΔrpoZ strain after a 24-h treatment at 40°C. (PDF) [file pone.0112599.s004.pdf]

Table S4. Genes down-regulated to half or less in the ΔrpoZ strain after a 24-h treatment at 40 °C.

ORF	FC*	P value	Function	Gene name (if assigned)	Functional category**
sll0450	-3.1	0.0000	cytochrome b subunit of nitric oxide reductase	*norB*	A
sll1713	-1.363	0.0006	histidinol-phosphate aminotransferase	*hisC*	A
slr0387	-1.3	0.0149	cysteine desulfurase NifS	*nifS1*	A
slr0710	-1.06	0.0001	glutamate dehydrogenase (NADP+)	*gdhA*	A
sll1185	-1.137	0.0037	coproporphyrinogen III oxidase, aerobic (oxygen-dependent)	*hemF*	B
slr0426	-1.367	0.0003	GTP cyclohydrolase I	*folE*	B
slr0623	-1.33	0.0024	thioredoxin	*trxA*	B
slr0636	-1.01	0.0044	probable cobalamin [5'-phosphate] synthase		B
slr0749	-2.84	0.0000	light-independent protochlorophyllide reductase iron protein subunit ChlL	*chlL*	B
slr0750	-1.433	0.0101	light-independent protochlorophyllide reductase subunit ChlN	*chlN*	B
slr0772	-2.143	0.0026	light-independent protochlorophyllide reductase subunit ChlB	*chlB*	B
slr0994	-1.063	0.0071	lipoate-protein ligase B	*lipB*	B
slr1239	-1.133	0.0317	pyridine nucleotide transhydrogenase alpha subunit	*pntA*	B
slr1072	-1.047	0.0007	GDP-D-mannose dehydratase	*rfbD, yefA*	C
slr1351	-1.513	0.0017	UDP-N-acetylmuramoylalanyl-D-glutamyl-2 6-diaminopimelate--D-alanyl-D-alanine ligase	*murF*	C
sll1384	-1.263	0.0041	similar to DnaJ protein	*dnaJ*	D
sll1533	-1.997	0.0140	twitching mobility protein	*pilT2*	D
sll1615	-1.08	0.0485	thiophen and furan oxidation protein	*trmE, thdF*	D
slr0161	-1.247	0.0019	twitching motility protein PilT	*pilT1*	D
slr0162	-1.04	0.0047	a part of pilC, pilin biogenesis protein, required for twitching motility	*pilC*	D
slr0427	-1.157	0.0008	putative competence-damage protein	*psbA2*	D
slr0950	-1.02	0.0179	hemolysin-like protein		D
slr2075	-1.857	0.0001	10kD chaperonin	*groES*	D
slr2076	-1.867	0.0001	60kD chaperonin	*groEL1*	D
sll0842	-1.257	0.0006	neopullulanase	*nplT*	E
sll0542	-1.013	0.0006	acetyl-coenzyme A synthetase	*acs*	F
sll0573	-3.383	0.0022	carbamate kinase	*arc*	F
sll0593	-1.263	0.0006	glucokinase	*glk*	F
sll1479	-1.867	0.0003	6-phosphogluconolactonase	*devB, pgl*	F
slr0301	-2.673	0.0041	phosphoenolpyruvate synthase	*ppsA*	F
slr1705	-2.02	0.0044	aspartoacylase	*aspA*	F
slr2132	-1.003	0.0047	phosphotransacetylase	*pta*	F
sll0330	-1.007	0.0051	sepiapterine reductase	*fabG*	G
sll1441	-1.337	0.0303	acyl-lipid desaturase (omega-3)	*desB*	G
sll1655	-2	0.0002	similar to biotin [acetyl-CoA-carboxylase] ligase	*birA*	G
sll0741	-1.75	0.0033	pyruvate flavodoxin oxidoreductase	*nifJ, Pfo*	H
sll1321	-1.007	0.0053	hypothetical protein	*atp1*	H
sml0004	-1.117	0.0075	cytochrome b6-f complex subunit VIII	*petN*	H
smr0001	-1.003	0.0098	photosystem II PsbT protein	*psbT*	H
smr0005	-1.123	0.0026	photosystem I subunit XII	*psaM*	H
ssl3044	-1.707	0.0190	probable ferredoxin		H
slr1164	-1.27	0.0006	ribonucleotide reductase subunit alpha	*nrdA, dnaF*	I
sll0485	-1.08	0.0223	two-component response regulator NarL subfamily	*rre30*	J

ORF	FC*	P value	Function	Gene name (if assigned)	Functional category**
sll1003	-1.317	0.0005	two-component sensor histidine kinase	*hik13*	J
sll1286	-2.137	0.0017	transcriptional regulator		J
sll1334	-1.107	0.0026	two-component sensor histidine kinase		J
sll1626	-1.813	0.0005	LexA repressor	*lexA*	J
slr0449	-1.347	0.0064	probable transcriptional regulator	*dnr*	J
slr0473	-1.893	0.0018	cyanobacterial phytochrome 1, two-component sensor histidine kinase	*hik3*	J
slr0474	-2.34	0.0001	two-component response regulator	*rre27*	J
slr1214	-1.977	0.0033	two-component response regulator	*rre15*	J
slr1584	-1.167	0.0030	two-component transcription regulator	*rre38*	J
sll0485	-1.017	0.0184	two-component hybrid sensor and regulator	*hik21*	J
sll0709	-1.427	0.0064	putative endonuclease	*llaI.2*	K2
slr0790	-1.447	0.0003	similar to ultraviolet light resistance protein B	*umuC*	K2
slr1564	-1.247	0.0002	group 3 RNA polymerase sigma factor	*sigF*	L
sll0825	-1.017	0.0000	polyA polymerase	*pcnB*	M
sll0830	-2.167	0.0006	elongation factor EF-G	*Fus*	M
sll1198	-1.79	0.0037	tRNA (guanine-N1)-methyltransferase	*trmD*	M
sll1712	-1.503	0.0009	DNA binding protein HU		M
sll1744	-1.327	0.0003	50S ribosomal protein L1	*rpl1*	M
sll1745	-1.793	0.0001	50S ribosomal protein L10	*rpl10*	M
sll1746	-1.563	0.0002	50S ribosomal protein L12	*rpl12*	M
slr0955	-1.063	0.0282	probable tRNA/rRNA methyltransferase		M
sll0771	-1.437	0.0007	glucose transport protein	*glcP*	N
sll1206	-1.24	0.0089	ferric aerobactin receptor, FhuA homolog	*iutA*	N
slr0681	-1.797	0.0010	probable sodium/calcium exchanger protein		N
slr1890	-1.04	0.0011	bacterioferritin	*bfrB*	N
slr2107	-1.153	0.0040	probable polysaccharide ABC transporter permease protein	*rfbA*	N
sll0222	-1.08	0.0063	putative purple acid phosphatase	*phoA*	O
sll0947	-1.913	0.0002	light repressed protein A homolog	*lrtA*	O
sll1308	-1.073	0.0046	probable oxidoreductase		O
sll1491	-1.04	0.0016	periplasmic WD-repeat protein		O
sll1534	-1.477	0.0006	probable glycosyltransferase	*rfbZ*	O
sll1723	-1.43	0.0055	probable glycosyltransferase		O
slr1019	-1.397	0.0010	phenazine biosynthetic protein PhzF homolog		O
slr1065	-1.063	0.0091	probable glycosyltransferase		O
slr1523	-1.003	0.0172	putative transposase		O
slr1888	-1.203	0.0001	4-hydroxybutyrate coenzyme A transferase.		O
sml0010	-1.137	0.0300	putative transposase		O
ssr1789	-1.017	0.0006	CAB/ELIP/HLIP-related protein HliD	*hliD*	O
ssr2078	-1.12	0.0038	putative transposase [ISY802b(partial copy): 1384736 - 1385513]	*ISY802b*	O
ssr2227	-1.76	0.0033	putative transposase		O
ssr2595	-1.54	0.0008	high light-inducible polypeptide HliB, CAB/ELIP/HLIP superfamily	*hliB*	O
sll0023	-1.147	0.0159	hypothetical protein		P
sll0051	-1.7	0.0010	hypothetical protein		P
sll0253	-2.85	0.0003	hypothetical protein		P
sll0283	-1.957	0.0001	hypothetical protein		P

ORF	FC*	P value	Function	Gene name (if assigned)	Functional category**
sll0360	-1.12	0.0036	hypothetical protein		P
sll0412	-1.35	0.0018	hypothetical protein		P
sll0451	-2.613	0.0005	hypothetical protein		P
sll0543	-1.393	0.0002	hypothetical protein		P
sll0572	-1.697	0.0196	hypothetical protein		P
sll0611	-1.163	0.0003	hypothetical protein		P
sll0615	-1.183	0.0092	hypothetical protein		P
sll0905	-1.133	0.0011	hypothetical protein	*maf*	P
sll1348	-1.26	0.0004	hypothetical protein		P
sll1358	-1.317	0.0001	putative oxalate decarboxylase, periplasmic protein	*mncA*	P
sll1473	-1.013	0.0023	a part of phytochrome-like sensor histidine kinase gene (disrupted by insertion of IS)	*ccaS,hik32*	P
sll1500	-1.747	0.0068	hypothetical protein		P
sll1516	-1.517	0.0017	hypothetical protein		P
sll1654	-1.923	0.0003	hypothetical protein	*usp*	P
sll1722	-1.94	0.0258	hypothetical protein		P
sll1965	-1.227	0.0044	hypothetical protein		P
slr0053	-1.03	0.0058	hypothetical protein		P
slr0105	-1.03	0.0110	hypothetical protein		P
slr0144	-2.25	0.0009	hypothetical protein		P
slr0146	-1.47	0.0060	hypothetical protein		P
slr0147	-1.77	0.0000	hypothetical protein		P
slr0148	-1.49	0.0001	hypothetical protein	*fdx*	P
slr0179	-1.403	0.0068	hypothetical protein		P
slr0244	-1.42	0.0001	hypothetical protein	*usp1*	P
slr0249	-1.013	0.0012	hypothetical protein		P
slr0359	-1.053	0.0079	hypothetical protein	*syn-lov*	P
slr0554	-1.233	0.0014	hypothetical protein		P
slr0680	-2.123	0.0014	hypothetical protein		P
slr0686	-1.01	0.0049	hypothetical protein		P
slr0789	-1.633	0.0003	hypothetical protein		P
slr0869	-1.453	0.0168	hypothetical protein		P
slr0878	-1.01	0.0011	hypothetical protein		P
slr0888	-3.42	0.0001	hypothetical protein		P
slr0959	-1.327	0.0009	hypothetical protein		P
slr1068	-1.467	0.0058	hypothetical protein		P
slr1069	-1.64	0.0173	hypothetical protein		P
slr1161	-1.107	0.0110	hypothetical protein		P
slr1241	-1.053	0.0431	hypothetical protein		P
slr1259	-1.997	0.0001	hypothetical protein		P
slr1260	-1.67	0.0001	hypothetical protein		P
slr1438	-2.41	0.0001	hypothetical protein		P
slr1444	-1.13	0.0001	hypothetical protein		P
slr1506	-1.3	0.0193	hypothetical protein		P
slr1692	-1.143	0.0029	hypothetical protein		P

ORF	FC*	P value	Function	Gene name (if assigned)	Functional category**
slr1704	-3.857	0.0000	hypothetical protein		P
slr1840	-1.377	0.0040	hypothetical protein	*synGK*	P
slr1851	-2.507	0.0018	hypothetical protein	*rfrH*	P
slr1957	-1.873	0.0025	hypothetical protein		P
slr1963	-2.007	0.0004	water-soluble carotenoid protein	*ocp*	P
slr1971	-1.46	0.0026	hypothetical protein		P
slr2031	-1.227	0.0026	putative PP2C-type protein phosphatase	*rsbU*	P
slr2052	-1.373	0.0021	hypothetical protein		P
ssl1046	-1.35	0.0003	hypothetical protein		P
ssl1911	-6.29	0.0000	glutamine synthetase inactivating factor IF7	*gifA*	P
ssl3573	-1.433	0.0025	hypothetical protein		P
ssr0692	-3.94	0.0000	hypothetical protein		P
ssr1251	-3.303	0.0002	hypothetical protein		P
ssr1562	-2.317	0.0003	hypothetical protein		P
ssr2062	-1.793	0.0002	hypothetical protein		P
ssr2802	-1.053	0.0151	hypothetical protein		P
sll0376	-1.273	0.0062	unknown protein		Z
sll0444	-1.017	0.0004	unknown protein		Z
sll0723	-1.017	0.0055	unknown protein		Z
sll0775	-1.817	0.0035	unknown protein		Z
sll1009	-1.837	0.0017	unknown protein	*frpC*	Z
sll1236	-1.847	0.0017	unknown protein		Z
sll1239	-3.333	0.0001	unknown protein		Z
sll1240	-3.837	0.0013	unknown protein		Z
sll1241	-3.167	0.0002	unknown protein		Z
sll1429	-1.267	0.0483	unknown protein		Z
sll1515	-5.023	0.0000	glutamine synthetase inactivating factor IF17	*gifB*	Z
sll1583	-1.05	0.0079	unknown protein	*ligA*	Z
sll1882	-1.207	0.0029	unknown protein		Z
slr0069	-1.11	0.0014	unknown protein		Z
slr0145	-1.903	0.0006	unknown protein		Z
slr0226	-1.637	0.0062	unknown protein		Z
slr0579	-1.657	0.0014	unknown protein		Z
slr0581	-2.363	0.0001	unknown protein		Z
slr0582	-1.1	0.0010	unknown protein		Z
slr0587	-2.09	0.0006	unknown protein		Z
slr0593	-1.123	0.0457	cAMP binding membrane protein	*samp*	Z
slr0616	-1.86	0.0005	unknown protein	*mvrA*	Z
slr0868	-1.09	0.0056	unknown protein		Z
slr1070	-1.543	0.0028	unknown protein		Z
slr1071	-1.28	0.0001	unknown protein		Z
slr1073	-1.163	0.0132	unknown protein		Z
slr1074	-1.21	0.0197	unknown protein		Z
slr1258	-1.03	0.0319	unknown protein		Z
slr1437	-2.74	0.0001	unknown protein		Z

ORF	FC*	P value	Function	Gene name (if assigned)	Functional category**
slr1544	-1.497	0.0017	unknown protein		Z
slr1667	-2.053	0.0271	hypothetical protein (target gene of sycrp1)	*cccS*	Z
slr1670	-1.563	0.0016	unknown protein		Z
slr1788	-1.08	0.0138	unknown protein		Z
slr1862	-2.613	0.0058	unknown protein		Z
slr1958	-1.573	0.0039	unknown protein		Z
ssl0318	-1.567	0.0072	unknown protein		Z
ssl1326	-1.04	0.0058	unknown protein		Z
ssl1533	-1.19	0.0010	unknown protein		Z
ssl2384	-1.857	0.0005	unknown protein		Z
ssl2653	-1.377	0.0015	unknown protein		Z
ssl2891	-1.427	0.0156	unknown protein		Z
ssr0693	-1.17	0.0096	unknown protein		Z
ssr1038	-4.717	0.0001	unknown protein		Z
ssr2153	-1.66	0.0052	unknown protein		Z
ssr2194	-3.773	0.0001	unknown protein		Z
ssr3129	-2.56	0.0019	unknown protein		Z

*FC: $\log_2$ of fold change.

**The categories are listed according to Cyanobase (see Fig. 2).
