# Supplementary material for: The ω Subunit of RNA Polymerase Is Essential for Thermal Acclimation of the Cyanobacterium Synechocystis Sp. PCC 6803
Source: PLoS One. 2014 Nov 11;9(11):e112599. doi: 10.1371/journal.pone.0112599 (PMC4227741; doi:10.1371/journal.pone.0112599)
Supplement: Table S5 — List of genes included in Fig. 3 and their expression data. (PDF) [file pone.0112599.s005.pdf]

Table S5. List of genes included in Fig. 3 and their expression data.

ORF	ΔrpoZ$_{40}$/ΔrpoZ$_{32}$		CS$_{40}$/CS$_{32}$		ΔrpoZ$_{32}$/CS$_{32}$		Description	Gene name	Functional category**	
	FC*	P-value	FC*	P-value	FC*	P-value				
sll1454	3.99	0.0001	0.57	0.5434	-2.02	0.0011	ferredoxin-nitrate reductase	narB	A	Amino acid biosynthesis
slr0898	3.91	0.0000	2.05	0.1081	-0.46	0.5611	ferredoxin--nitrite reductase	nirA	A	
slr0899	3.91	0.0007	2.04	0.1595	-0.22	0.7726	cyanate lyase	cynS	A	
sll0784	2.42	0.0007	2.14	0.0574	1.30	0.0036	nitrilase	merR	A	
slr0288	1.79	0.0004	2.65	0.0423	1.42	0.0355	glutamate--ammonia ligase	glnN	A	
slr1756	1.79	0.0000	2.01	0.0127	-0.11	0.8037	glutamate--ammonia ligase	glnA	A	
sll1499	1.46	0.0056	-0.12	0.2601	-1.33	0.0019	ferredoxin-dependent glutamate synthase	glsF, gltS	A	
slr0963	1.39	0.0027	0.23	0.0051	-0.66	0.0128	ferredoxin-sulfite reductase	sir	A	
slr0077	1.15	0.0117	-0.75	0.1158	-1.62	0.0083	cysteine desulfurase	sufS, nifS	A	
slr0186	1.09	0.0018	0.25	0.5058	0.34	0.2925	2-isopropylmalate synthase	leuA	A	
sll1883	1.08	0.0014	0.46	0.0006	0.11	0.4035	arginine biosynthesis bifunctional protein ArgJ	argJ	A	
sll1688	0.95	0.0130	-1.02	0.0029	-1.99	0.0000	threonine synthase	thrC	A	
ssl0707	0.90	0.0125	1.42	0.0306	0.38	0.2612	nitrogen regulatory protein P-II	glnB	A	
slr1898	0.51	0.0010	1.27	0.0316	0.17	0.1016	N-acetylglutamate kinase	argB	A	
slr0710	-1.06	0.0001	-0.27	0.5090	0.55	0.0000	glutamate dehydrogenase (NADP+)	gdhA	A	
slr0387	-1.30	0.0149	0.25	0.4467	1.58	0.0020	cysteine desulfurase NifS	nifS1	A	
sll1713	-1.36	0.0006	-0.34	0.1838	0.38	0.1497	histidinol-phosphate aminotransferase	hisC	A	
sll0450	-3.10	0.0000	-2.39	0.0002	0.49	0.0937	cytochrome b subunit of nitric oxide reductase	norB	A	
slr0901	3.36	0.0003	1.45	0.1064	-0.60	0.0934	molybdopterin biosynthesis protein A	moaA	B	Biosynthesis of cofactors, prosthetic groups, and carriers
slr0900	3.35	0.0001	1.66	0.1243	-0.41	0.3580	molybdopterin biosynthesis MoeA protein	moeA	B	
ssr1527	2.53	0.0016	0.61	0.4921	-0.35	0.3384	probable molybdopterin [MPT] converting factor, subunit 1	moaD	B	
slr0902	2.10	0.0006	0.90	0.2499	-0.20	0.4043	molybdenum cofactor biosynthesis protein C, fused to MobA	moaC	B	
slr1254	1.74	0.0082	-0.58	0.0923	-1.80	0.0043	phytoene dehydrogenase (phytoene desaturase)	crtP	B	
ssr2061	1.38	0.0009	0.45	0.0109	-0.77	0.0014	glutaredoxin	grxA, grxC	B	
slr1300	1.15	0.0000	-0.46	0.0304	-0.92	0.0004	similar to 2-octaprenyl-6-methoxyphenol hydroxylase	ubiH, visB	B	
slr0839	1.12	0.0000	-0.15	0.1423	-1.19	0.0000	ferrochelatase	hemH, scpA	B	
slr1238	1.02	0.0258	-0.26	0.2485	-1.06	0.0109	glutathione synthetase	gshB	B	
slr1171	1.01	0.0143	0.02	0.8533	0.02	0.9329	glutathione peroxidase-like NADPH peroxidase,glutathione peroxidase	gpx1	B	
slr1434	-0.22	0.4703	-1.10	0.0068	-1.11	0.0215	pyridine nucleotide transhydrogenase beta subunit	pntB	B	
slr0636	-1.01	0.0044	-0.12	0.2574	0.54	0.0065	probable cobalamin [5'-phosphate] synthase		B	
slr0994	-1.06	0.0071	-0.26	0.0786	0.55	0.0271	lipoate-protein ligase B	lipB	B	
slr1239	-1.13	0.0317	-0.01	0.9667	0.76	0.0494	pyridine nucleotide transhydrogenase alpha subunit	pntA	B	
sll1185	-1.14	0.0037	-0.38	0.1347	0.27	0.3247	coproporphyrinogen III oxidase, aerobic (oxygen-dependent)	hemF	B	
slr0623	-1.33	0.0024	0.86	0.0381	2.07	0.0014	thioredoxin	trxA	B	
slr0426	-1.37	0.0003	-1.02	0.0405	-0.16	0.3054	GTP cyclohydrolase I	folE	B	
slr0750	-1.43	0.0101	-0.53	0.0713	0.78	0.0297	light-independent protochlorophyllide reductase	chlN	B	

ORF	ΔrpoZ$_{40}$/ΔrpoZ$_{32}$		CS$_{40}$/CS$_{32}$		ΔrpoZ$_{32}$/CS$_{32}$		Description	Gene name	Functional category**	
	FC*	P-value	FC*	P-value	FC*	P-value				
							subunit ChlN			
slr0772	-2.14	0.0026	0.86	0.1816	2.29	0.0016	light-independent protochlorophyllide reductase subunit ChlB	chlB	B	
slr0749	-2.84	0.0000	-1.11	0.0042	1.33	0.0017	light-independent protochlorophyllide reductase iron protein subunit ChlL	chlL	B	
sll1271	1.64	0.0100	1.39	0.0936	-0.37	0.2311	probable porin; major outer membrane protein		C	Cell envelope
slr0993	1.38	0.0037	0.25	0.3606	-1.02	0.0095	putative peptidase	nlpD	C	
slr0827	1.10	0.0002	0.82	0.0003	-0.30	0.0087	alanine racemase	dal, alr	C	
slr1064	-0.37	0.4127	-1.66	0.0008	-0.68	0.1318	probable glycosyltransferase	rfbU, mtfA	C	
sll1724	-0.41	0.2345	-1.09	0.0243	-0.15	0.7147	probable glycosyltransferase	icsA	C	
slr0984	-0.52	0.0334	-1.37	0.0001	-0.63	0.0103	CDP-glucose 4,6-dehydratase	rfbG	C	
slr1072	-1.05	0.0007	-1.11	0.0001	-0.06	0.5731	GDP-D-mannose dehydratase	rfbD, yefA	C	
slr1351	-1.51	0.0017	-1.01	0.0415	0.03	0.7670	UDP-N-acetylmuramoylalanyl-D-glutamyl-2 6-diaminopimelate--D-alanyl-D-alanine ligase	murF	C	
sll1294	2.48	0.0003	0.71	0.0327	-1.73	0.0002	methyl-accepting chemotaxis protein	taxD2	D	Cellular processes
sll1666	1.51	0.0017	0.53	0.0053	-0.49	0.0419	DnaJ-like protein	dnaJ	D	
slr1604	1.34	0.0146	-0.31	0.1384	-0.99	0.0191	cell division protein FtsH	ftsH3,	D	
slr0228	1.04	0.0007	-0.50	0.0080	-1.00	0.0001	cell division protein FtsH	ftsH2	D	
sll1514	0.91	0.0233	1.45	0.0400	1.92	0.0000	16.6 kDa small heat shock protein, molecular chaperone	hspA	D	
sll1695	0.86	0.0161	2.19	0.0001	1.00	0.0053	pilin polypeptide PilA2	pilA2	D	
sll1694	0.77	0.0099	1.88	0.0002	0.66	0.0249	pilin polypeptide PilA1	pilA1	D	
ssl2922	0.31	0.4929	-1.15	0.0251	-0.74	0.1671	similar to virulence-associated protein VapB	vapB	D	
sll0430	-0.81	0.0658	-1.73	0.0050	-1.22	0.0225	HtpG, heat shock protein 90	htpG	D	
sml0009	-0.21	0.5399	-1.14	0.0227	-0.50	0.2729	similar to virulence-associated protein VapC		D	
slr0950	-1.02	0.0179	0.00	0.9954	0.26	0.4142	hemolysin-like protein		D	
slr0162	-1.04	0.0047	0.18	0.3499	1.46	0.0004	a part of pilC	pilC	D	
sll1615	-1.08	0.0485	-0.07	0.6589	1.09	0.0184	thiophen and furan oxidation protein	trmE, thdF	D	
slr0427	-1.16	0.0008	-1.00	0.0023	-0.30	0.1512	putative competence-damage protein	psbA2	D	
slr0161	-1.25	0.0019	-0.07	0.7724	1.58	0.0001	twitching motility protein PilT	pilT1	D	
sll1384	-1.26	0.0041	-0.77	0.0003	0.34	0.0128	similar to DnaJ protein	dnaJ	D	
slr2075	-1.86	0.0001	-1.08	0.0106	-0.46	0.0095	10kD chaperonin	groES	D	
slr2076	-1.87	0.0001	-0.73	0.0727	-0.16	0.2075	60kD chaperonin	groEL1	D	
sll1533	-2.00	0.0140	0.24	0.4217	1.96	0.0000	twitching mobility protein	pilT2	D	
slr1857	2.32	0.0003	0.17	0.3666	-1.49	0.0011	isoamylase	glgX	E	Central intermediary metabolism
slr1367	1.68	0.0036	0.00	0.9861	-0.99	0.0080	glycogen phosphorylase	glgP2	E	
sll1676	1.38	0.0003	0.43	0.0102	-0.41	0.0124	4-alpha-glucanotransferase	malQ	E	
sll0220	1.12	0.0006	0.15	0.5762	-0.65	0.0582	L-glutamine:D-fructose-6-P amidotransferase	glmS	E	
sll0842	-1.26	0.0006	0.12	0.2629	1.23	0.0001	Neopullulanase	nplT	E	
slr1289	1.77	0.0002	1.81	0.0097	0.74	0.0011	isocitrate dehydrogenase (NADP+)	Icd	F	Energy metabolism
slr0293	1.71	0.0022	-1.17	0.0007	-2.68	0.0000	glycine dehydrogenase	gcvP	F	
sll1498	1.62	0.0001	0.39	0.1739	-0.34	0.0148	carbamoyl-phosphate synthase small chain	carA, pyrA	F	
sll0018	1.57	0.0000	0.58	0.0127	-1.43	0.0001	fructose-bisphosphate aldolase, class II	fbaA, fda	F	

ORF	ΔrpoZ40/ΔrpoZ32		CS40/CS32		ΔrpoZ32/CS32		Description	Gene name	Functional category**	
	FC*	P-value	FC*	P-value	FC*	P-value				
sll1234	1.47	0.0030	-0.17	0.4295	-1.35	0.0013	adenosylhomocysteinase	ahcY	F	
sll1077	1.34	0.0039	0.94	0.0225	0.20	0.5074	agmatinase	speB2	F	
sll0404	1.26	0.0011	0.38	0.0280	-0.35	0.0479	glycolate oxidase subunit GlcD	glcD	F	
slr2132	-1.00	0.0047	-0.18	0.4713	1.20	0.0031	phosphotransacetylase	pta	F	
sll0542	-1.01	0.0006	0.30	0.0290	1.48	0.0000	acetyl-coenzyme A synthetase	acs	F	
sll0593	-1.26	0.0006	-0.38	0.0175	-0.03	0.7903	glucokinase	glk	F	
sll1479	-1.87	0.0003	-0.74	0.0083	0.95	0.0017	6-phosphogluconolactonase	devB, pgl	F	
slr1705	-2.02	0.0044	-1.26	0.0038	-0.52	0.0259	aspartoacylase	aspA	F	
slr0301	-2.67	0.0041	0.97	0.0047	3.94	0.0003	phosphoenolpyruvate synthase	ppsA	F	
sll0573	-3.38	0.0022	-0.70	0.0006	2.27	0.0025	carbamate kinase	arc	F	
slr0574	1.00	0.0003	-0.02	0.9625	-0.46	0.0975	cytochrome P450	cyp	G	Fatty acid, phospholipid and sterol metabolism
slr1350	0.00	1.0000	-1.15	0.0005	-1.65	0.0015	acyl-lipid desaturase (delta 12)	desA	G	
sll0330	-1.01	0.0051	-1.19	0.0470	0.51	0.1142	sepiapterine reductase	fabG	G	
sll1441	-1.34	0.0303	-2.40	0.0019	-0.14	0.7684	acyl-lipid desaturase (omega-3)	desB	G	
sll1655	-2.00	0.0002	0.48	0.2435	2.17	0.0001	similar to biotin [acetyl-CoA-carboxylase] ligase	birA	G	
ssl0452	2.54	0.0001	2.40	0.0709	0.14	0.6306	phycobilisome degradation protein NblA	nblA1	H	Photosynthesis and respiration
ssl0453	2.38	0.0001	1.81	0.1151	-0.26	0.2853	phycobilisome degradation protein NblA	nblA2	H	
ssl2559	2.26	0.0041	1.23	0.0023	-0.83	0.0412	ferredoxin		H	
sll1732	1.92	0.0017	-2.16	0.1705	-2.96	0.0001	NADH dehydrogenase subunit 5 (involved in low CO2-inducible, high affinity CO2 uptake)	ndhF3	H	
slr1281	1.70	0.0009	-0.42	0.5177	-1.70	0.0003	NADH dehydrogenase subunit I	ndhJ	H	
slr1279	1.56	0.0040	-0.25	0.6632	-1.42	0.0019	NADH dehydrogenase subunit 3	ndhC	H	
slr1280	1.47	0.0043	-0.35	0.5018	-1.31	0.0019	NADH dehydrogenase subunit NdhK	ndhK	H	
sll1733	1.36	0.0311	-1.94	0.2149	-1.91	0.0014	NADH dehydrogenase subunit 4 (involved in low CO2-inducible, high affinity CO2 uptake)	ndhD3	H	
slr0851	1.31	0.0007	1.20	0.0350	0.39	0.0750	type 2 NADH dehydrogenase	ndbA	H	
sll0519	1.30	0.0037	-0.69	0.1794	-1.89	0.0001	NADH dehydrogenase subunit 1	ndhA	H	
slr1380	1.30	0.0149	0.96	0.0672	-0.01	0.9789	quinol oxidase subunit II	cydB	H	
slr2059	1.28	0.0096	0.26	0.2880	-1.07	0.0030	iron-sulfur cluster binding protein homolog		H	
sll0520	1.21	0.0043	-0.63	0.2245	-1.85	0.0001	NADH dehydrogenase subunit NdhI	ndhI	H	
slr1643	1.11	0.0003	0.38	0.2630	0.19	0.0235	ferredoxin-NADP oxidoreductase	petH	H	
slr1379	1.11	0.0038	0.93	0.1075	0.20	0.2615	quinol oxidase subunit I	cydA	H	
sll1031	1.09	0.0470	-1.00	0.0967	-2.04	0.0017	carbon dioxide concentrating mechanism protein CcmM, putative carboxysome structural protein	ccmM, ndhR	H	
sll0247	0.76	0.0024	1.86	0.0128	0.78	0.0640	iron-stress chlorophyll-binding protein, homologous to psbC (CP43)	isiA	H	
ssr0390	0.15	0.4716	1.04	0.0447	-0.80	0.0113	photosystem I reaction center subunit X	psaK1	H	
slr2007	0.16	0.4948	-3.32	0.0016	-3.08	0.0002	NADH dehydrogenase subunit 4	ndhD5	H	
slr1291	0.06	0.4417	-1.87	0.0002	-2.49	0.0000	NADH dehydrogenase subunit 4	ndhD2	H	
sll0248	0.19	0.5445	1.07	0.0471	0.27	0.4813	flavodoxin	isiB	H	
slr2009	-0.01	0.9671	-1.74	0.0023	-1.33	0.0115	NADH dehydrogenase subunit 4	ndhD6	H	
smr0001	-1.00	0.0098	-0.10	0.6555	0.23	0.3651	photosystem II PsbT protein	psbT	H	
sll1321	-1.01	0.0053	0.06	0.8279	-0.44	0.0344	hypothetical protein	atp1	H	

ORF	ΔrpoZ$_{40}$/ΔrpoZ$_{32}$		CS$_{40}$/CS$_{32}$		ΔrpoZ$_{32}$/CS$_{32}$		Description	Gene name	Functional category**	
	FC*	P-value	FC*	P-value	FC*	P-value				
sml0004	-1.12	0.0075	-0.43	0.2331	0.00	0.9948	cytochrome b6-f complex subunit VIII	petN	H	
smr0005	-1.12	0.0026	0.26	0.3225	0.83	0.0216	photosystem I subunit XII	psaM	H	
ssl3044	-1.71	0.0190	-1.23	0.0069	0.74	0.0169	probable ferredoxin		H	
sll0741	-1.75	0.0033	-0.36	0.0598	2.48	0.0001	pyruvate flavodoxin oxidoreductase	nifJ, Pfo	H	
slr1164	-1.27	0.0006	-1.41	0.0000	-0.49	0.0022	ribonucleotide reductase subunit alpha	nrdA, dnaF	I	Purines, pyrimidines, nucleosides, nucleotides
sll1161	4.55	0.0001	1.71	0.0003	-0.87	0.0363	probable adenylate cyclase	cya3	J	Regulatory functions
slr1594	2.54	0.0010	0.68	0.3234	-0.68	0.0648	two-component response regulator	rre5	J	
sll1296	2.12	0.0003	0.63	0.0482	-1.25	0.0003	two-component hybrid sensor and regulator	hik39	J	
sll0782	2.00	0.0116	1.94	0.0204	0.73	0.2280	transcriptional regulator		J	
sll1291	1.70	0.0003	0.77	0.2070	-0.01	0.9681	two-component response regulator	rre12	J	
slr1860	1.54	0.0035	0.03	0.8947	-0.71	0.0168	carbon metabolisms regulatory protein IcfG	icfG	J	
sll1594	1.53	0.0380	-3.11	0.0003	-3.73	0.0001	ndhF3 operon transcriptional regulator, LysR family protein	ccmR, ndhR	J	
sll1330	1.40	0.0122	1.83	0.0197	1.01	0.0969	two-component system response regulator	rre37	J	
sll0567	1.24	0.0002	0.73	0.1222	-0.09	0.5920	ferric uptake regulation protein	fur	J	
sll1292	1.09	0.0006	0.69	0.0012	-0.52	0.0034	two-component response regulator	rre11	J	
sll1670	1.04	0.0046	0.05	0.8352	-0.64	0.0125	heat-inducible transcription repressor HrcA homolog	hrcA	J	
slr1285	-0.14	0.4534	-1.06	0.0159	-0.42	0.0749	two-component sensor histidine kinase	hik34	J	
slr1759	-0.41	0.1625	1.07	0.0372	0.95	0.0280	two-component hybrid sensor and regulator	hik14	J	
slr2098	-1.02	0.0184	0.05	0.7363	1.01	0.0031	two-component hybrid sensor and regulator	hik21	J	
sll0485	-1.08	0.0223	-0.17	0.7018	1.05	0.0070	two-component response regulator	rre30	J	
sll1334	-1.11	0.0026	-0.55	0.0014	0.28	0.1246	two-component sensor histidine kinase		J	
slr1584	-1.17	0.0030	0.90	0.0298	1.34	0.0063	two-component transcription regulator	rre38	J	
sll1003	-1.32	0.0005	0.21	0.4150	1.37	0.0014	two-component sensor histidine kinase	hik13	J	
slr0449	-1.35	0.0064	-0.28	0.0963	1.38	0.0012	probable transcriptional regulator	dnr	J	
sll1626	-1.81	0.0005	-1.33	0.0082	0.66	0.0334	LexA repressor	lexA	J	
slr0473	-1.89	0.0018	-0.33	0.2076	1.78	0.0006	cyanobacterial phytochrome 1, two-component sensor histidine kinase	hik35	J	
slr1214	-1.98	0.0033	-1.09	0.0143	0.04	0.9031	two-component response regulator PatA subfamily	rre15	J	
sll1286	-2.14	0.0017	-0.71	0.0019	1.01	0.0108	transcriptional regulator		J	
slr0474	-2.34	0.0001	-0.39	0.2692	2.08	0.0001	two-component response regulator	rre27	J	
slr1322	1.42	0.0003	-0.37	0.0905	-1.71	0.0000	putative modulator of DNA gyrase; TldD		K2	DNA replication, restriction, modification, recombination, and repair
slr1543	0.72	0.0006	1.00	0.0123	-0.09	0.0883	DNA-damage-inducible protein F		K2	
sll1772	-0.41	0.3201	-1.31	0.0236	-0.71	0.1995	DNA mismatch repair protein MutS	mutS	K2	
sll0709	-1.43	0.0064	-0.69	0.0356	0.50	0.1074	putative endonuclease	llaI.2	K2	
slr0790	-1.45	0.0003	-1.47	0.0440	-1.07	0.0081	similar to ultraviolet light resistance protein B	umuC	K2	
slr1859	1.91	0.0089	0.53	0.0209	-0.81	0.0626	anti-sigma f factor antagonist		L	Transcription
slr1856	1.72	0.0027	-0.09	0.6625	-1.31	0.0044	phosphoprotein substrate of icfG gene cluster	icfG	L	

ORF	ΔrpoZ40/ΔrpoZ32		CS40/CS32		ΔrpoZ32/CS32		Description	Gene name	Functional category**	
	FC*	P-value	FC*	P-value	FC*	P-value				
sll1787	1.34	0.0085	-0.24	0.1674	-0.77	0.0380	RNA polymerase beta subunit	rpoB	L	
sll2012	1.30	0.0026	-0.68	0.1441	-1.57	0.0009	group2 RNA polymerase sigma factor SigD	sigD	L	
slr1861	1.20	0.0026	0.01	0.9361	-0.36	0.1428	probable sigma regulatory factor		L	
slr0653	0.13	0.5248	-1.12	0.0001	-1.19	0.0004	principal RNA polymerase sigma factor SigA	sigA	L	
slr0083	0.19	0.6247	-1.43	0.0009	-1.30	0.0149	RNA helicase Light	crhR	L	
slr1912	1.13	0.0624	1.67	0.0022	-0.03	0.9215	putative PP2C-type protein phosphatase		L	
slr1564	-1.25	0.0002	-0.09	0.3932	0.86	0.0002	group 3 RNA polymerase sigma factor	sigF, rpoF	L	
sll2009	1.84	0.0003	0.74	0.0312	0.21	0.2118	processing protease	prp2	M	Translation
sll2008	1.78	0.0002	1.08	0.0154	0.73	0.0013	processing protease	prp1	M	
slr0008	1.02	0.0026	-0.26	0.0138	-0.97	0.0004	carboxyl-terminal processing protease	ctpA	M	
slr1204	1.00	0.0130	0.80	0.0596	0.51	0.0348	protease	degP	M	
slr1592	0.61	0.0345	-2.29	0.0002	-2.55	0.0000	probable pseudouridine synthase		M	
sll0555	-0.64	0.1072	-1.26	0.0256	-0.45	0.1924	methionine aminopeptidase	map-3, mapC	M	
sll0825	-1.02	0.0000	0.26	0.2896	0.66	0.0054	polyA polymerase	pcnB	M	
slr0955	-1.06	0.0282	-0.99	0.0747	-0.32	0.3624	probable tRNA/rRNA methyltransferase		M	
sll1744	-1.33	0.0003	-0.63	0.0912	-0.09	0.7559	50S ribosomal protein L1	rpl1	M	
sll1712	-1.50	0.0009	-0.08	0.3983	0.70	0.0050	DNA binding protein HU		M	
sll1746	-1.56	0.0002	-0.04	0.7928	0.04	0.7739	50S ribosomal protein L12	rpl12	M	
sll1198	-1.79	0.0037	1.73	0.0040	3.61	0.0000	tRNA (guanine-N1)-methyltransferase	trmD	M	
sll1745	-1.79	0.0001	-0.05	0.7622	0.15	0.3069	50S ribosomal protein L10	rpl10	M	
sll0830	-2.17	0.0006	0.73	0.0055	2.34	0.0000	elongation factor EF-G	fus	M	
sll1451	4.70	0.0001	1.78	0.1831	-1.88	0.0428	nitrate/nitrite transport system permease protein	nrtB	N	Transport and binding proteins
sll1453	4.50	0.0001	1.28	0.3155	-2.17	0.0068	nitrate/nitrite transport system ATP-binding protein	nrtD	N	
sll1450	4.37	0.0000	1.57	0.2362	-1.92	0.0301	nitrate/nitrite transport system substrate-binding protein	nrtA	N	
sll1452	4.30	0.0002	1.38	0.3084	-1.90	0.0305	nitrate/nitrite transport system ATP-binding protein	nrtC	N	
sll1081	2.13	0.0016	1.81	0.0591	1.45	0.0019	ABC transport system permease protein		N	
sll0108	2.08	0.0005	2.23	0.0216	0.45	0.1823	ammonium/methylammonium permease	amt1	N	
sll1270	1.98	0.0121	1.95	0.0158	-0.39	0.4413	periplasmic substrate-binding and integral membrane protein of the ABC-type Bgt permease for basic amino acids and glutamine BgtB	bgtB	N	
slr0944	1.91	0.0004	1.03	0.0281	0.57	0.0255	multidrug-efflux transporter	arsB	N	
sll1598	1.70	0.0001	1.88	0.0116	1.21	0.0008	Mn transporter MntC	mntC	N	
slr1200	1.51	0.0005	3.12	0.0056	0.82	0.2086	urea transport system permease protein	urtB	N	
sll1599	1.47	0.0002	1.63	0.0150	0.91	0.0028	manganese transport system ATP-binding protein MntA	mntA	N	
slr1735	1.39	0.0007	1.13	0.0368	0.61	0.0354	ATP-binding subunit of the ABC-type Bgt permease for basic amino acids and glutamine	bgtA	N	
sll1482	1.37	0.0012	-0.18	0.0333	-1.28	0.0003	ABC transporter permease protein		N	
sll0536	1.36	0.0081	2.09	0.0150	0.95	0.0326	probable potassium channel protein	kchX	N	
sll1017	1.30	0.0002	2.17	0.0362	0.29	0.0943	ammonium/methylammonium permease	amt2	N	

ORF	ΔrpoZ$_{40}$/ΔrpoZ$_{32}$		CS$_{40}$/CS$_{32}$		ΔrpoZ$_{32}$/CS$_{32}$		Description	Gene name	Functional category**	
	FC*	P-value	FC*	P-value	FC*	P-value				
slr0447	1.30	0.0020	1.54	0.1051	-0.40	0.1508	periplasmic protein, ABC-type urea transport system substrate-binding protein	urtA	N	
sll0834	1.28	0.0033	-0.19	0.2442	-1.10	0.0032	low affinity sulfate transporter	bicA	N	
sll1082	1.25	0.0003	0.92	0.1305	0.82	0.0202	ABC transport system ATP-binding protein		N	
sll1180	1.09	0.0200	-0.06	0.4683	-1.17	0.0054	toxin secretion ABC transporter ATP-binding protein	hlyB	N	
sll1481	1.02	0.0011	-0.06	0.7867	-0.67	0.0076	ABC-transporter membrane fusion protein		N	
sll0764	0.97	0.0101	1.34	0.0055	0.02	0.9097	urea transport system ATP-binding protein	urtD	N	
slr1488	0.81	0.0013	1.25	0.0006	-0.01	0.9331	multidrug resistance family ABC transporter		N	
slr1201	0.74	0.0162	1.87	0.0137	0.44	0.2068	urea transport system permease protein	urtC	N	
slr1318	0.30	0.0277	1.11	0.0012	0.03	0.8749	iron(III) dicitrate transport system ATP-binding protein	fecE	N	
sll1406	0.16	0.4692	1.69	0.0019	0.35	0.1657	ferrichrome-iron receptor	fhuA	N	
sll1404	0.37	0.1206	2.10	0.0043	0.51	0.2289	biopolymer transport ExbB protein homolog	exbB3	N	
sll0537	0.22	0.1080	1.04	0.0067	0.72	0.0006	ammonium/methylammonium permease	amt3	N	
sll1263	0.43	0.1829	-1.36	0.0028	-1.53	0.0011	cation efflux system protein		N	
slr0513	-0.32	0.3051	1.11	0.0008	0.67	0.0546	iron transport system substrate-binding protein, periplasmic protein	futA2	N	
slr1295	-0.31	0.2051	2.19	0.0001	1.21	0.0019	iron transport system substrate-binding protein	futA1	N	
slr1316	-0.09	0.6125	1.15	0.0084	0.43	0.0466	ABC-type iron(III) dicitrate transport system permease protein	fecC	N	
slr0096	-0.21	0.5551	1.07	0.0188	1.25	0.0044	low affinity sulfate transporter		N	
sll1405	-0.32	0.0331	1.97	0.0049	0.97	0.0285	biopolymer transport ExbD protein homolog	exbD	N	
slr1890	-1.04	0.0011	-0.39	0.0453	0.26	0.1371	bacterioferritin	bfrB	N	
slr2107	-1.15	0.0040	0.26	0.1929	1.05	0.0012	probable polysaccharide ABC transporter permease protein	rfbA	N	
sll1206	-1.24	0.0089	0.47	0.0362	1.48	0.0011	ferric aerobactin receptor, FhuA homolog	iutA	N	
sll0771	-1.44	0.0007	0.58	0.1957	1.34	0.0010	glucose transport protein	glcP	N	
slr0681	-1.80	0.0010	0.08	0.7726	2.03	0.0000	probable sodium/calcium exchanger protein		N	
slr2002	1.65	0.0045	1.66	0.0369	-0.10	0.7398	cyanophycin synthetase	cphA	O	Other categories
sll1079	1.57	0.0006	0.60	0.0934	-0.13	0.5167	putative hydrogenase expression/formation protein HypB	hypB	O	
sll1078	1.57	0.0069	0.99	0.0370	0.18	0.5683	putative hydrogenase expression/formation protein HypA	hypA2	O	
sll1297	1.55	0.0090	0.67	0.0324	-1.01	0.0088	probable dioxygenase, Rieske iron-sulfur component	pobA	O	
slr0756	1.48	0.0101	0.25	0.4643	-0.81	0.0334	circadian clock protein KaiA homolog	kaiA	O	
slr0946	1.47	0.0050	0.20	0.4764	-0.23	0.3393	arsenate reductase	arsC	O	
sll1154	1.47	0.0001	-0.21	0.1343	-0.96	0.0003	putative antibiotic efflux protein	norA	O	
slr0945	1.24	0.0104	0.47	0.1617	0.12	0.6529	arsenical resistance protein ArsH homolog	arsH	O	
sll0217	1.20	0.0010	-1.16	0.4533	-4.72	0.0002	flavoprotein	flv4	O	
slr0665	1.19	0.0011	0.59	0.1894	-0.08	0.5158	aconitate hydratase	acnB	O	
sll0550	1.18	0.0183	-0.33	0.2267	-1.09	0.0148	flavoprotein	flv3	O	
slr1498	1.05	0.0001	0.52	0.0416	0.36	0.0322	putative hydrogenase expression/formation protein HypD	hypD	O	

ORF	$\Delta rpoZ_{40}/\Delta rpoZ_{32}$		CS_{40}/CS_{32}		$\Delta rpoZ_{32}/CS_{32}$		Description	Gene name	Functional category**	
	FC*	P-value	FC*	P-value	FC*	P-value				
slr1675	1.02	0.0195	0.51	0.2775	1.16	0.0165	putative hydrogenase expression/formation protein HypA1	hypA1	O	
sll1621	1.01	0.0304	0.98	0.0787	-0.03	0.9251	AhpC/TSA family protein	type II prx	O	
slr1077	-0.64	0.0234	-1.20	0.0000	-0.56	0.0011	probable glycosyltransferase	gumH	O	
slr0067	-0.66	0.0062	-1.23	0.0001	-0.73	0.0001	MRP protein homolog	mrp	O	
ssr1789	-1.02	0.0006	-0.89	0.0014	-0.08	0.4975	High light-inducible HliD	hliD	O	
sll0222	-1.08	0.0063	-1.04	0.0155	-0.19	0.4895	putative purple acid phosphatase	phoA	O	
ssr2078	-1.12	0.0038	0.47	0.0346	1.84	0.0001	putative transposase [ISY802b(partial copy): 1384736 - 1385513]	ISY802b	O	
sll1534	-1.48	0.0006	0.05	0.9207	1.82	0.0009	probable glycosyltransferase	rfbZ	O	
ssr2595	-1.54	0.0008	-0.72	0.2077	0.54	0.0477	high light-inducible HliB,	hliB	O	
sll0947	-1.91	0.0002	0.28	0.1280	2.18	0.0000	light repressed protein A homolog	lrtA	O	
sll1159	6.08	0.0000	2.86	0.0000	-1.56	0.0011	probable bacterioferritin comigratory protein		O	
slr1853	2.09	0.0002	0.50	0.1662	-0.51	0.1432	carboxymuconolactone decarboxylase		O	
sll1305	1.71	0.0016	-0.09	0.7368	-1.15	0.0104	probable hydrolase		O	
slr0626	1.04	0.0003	0.47	0.0576	-0.08	0.6987	probable glycosyltransferase		O	
slr0541	1.01	0.0103	0.29	0.0744	0.54	0.0221	probable amidotransferase		O	
sll1407	0.93	0.0389	1.30	0.0038	-0.45	0.1439	probable methyltransferase		O	
slr1410	0.79	0.1341	-1.04	0.0080	-1.49	0.0088	periplasmic WD-repeat protein		O	
slr1063	-0.54	0.2553	-1.57	0.0016	-0.29	0.4561	probable glycosyltransferase		O	
slr1610	-0.51	0.0005	-1.12	0.0297	-0.34	0.1257	putative C-3 methyl transferase		O	
slr1076	-0.88	0.0090	-1.29	0.0000	-0.33	0.0531	probable glycosyltransferase		O	
slr1523	-1.00	0.0172	-0.66	0.0191	1.50	0.0022	putative transposase		O	
sll1491	-1.04	0.0016	0.09	0.7791	0.68	0.0055	periplasmic WD-repeat protein		O	
slr1065	-1.06	0.0091	-1.49	0.0003	0.03	0.8953	probable glycosyltransferase		O	
sll1308	-1.07	0.0046	0.31	0.0860	0.33	0.0908	probable oxidoreductase		O	
sml0010	-1.14	0.0300	0.96	0.0064	1.14	0.0159	putative transposase		O	
slr1888	-1.20	0.0001	-0.34	0.0144	1.24	0.0000	4-hydroxybutyrate coenzyme A transferase.		O	
slr1019	-1.40	0.0010	-1.49	0.0454	-2.19	0.0000	phenazine biosynthetic protein PhzF homolog		O	
sll1723	-1.43	0.0055	-0.62	0.1816	0.67	0.1463	probable glycosyltransferase		O	
ssr2227	-1.76	0.0033	0.72	0.0172	1.93	0.0014	putative transposase		O	
slr1593	1.50	0.0001	0.94	0.0347	0.34	0.1164	hypothetical protein	ylmD	P	Hypothetical
slr1276	1.23	0.0028	-0.12	0.5459	-1.29	0.0011	hypothetical protein	pilO	P	
sll1314	1.21	0.0020	0.09	0.7460	-1.31	0.0011	putative C4-dicarboxylase binding protein, periplasmic protein	dctP	P	
sll1734	1.17	0.0098	-1.68	0.2766	-1.11	0.0115	protein involved in low CO2-inducible, high affinity CO2 uptake	cupA	P	
sll1541	1.16	0.0210	-0.65	0.0390	-1.06	0.0105	hypothetical protein	syc2	P	
slr0076	1.14	0.0259	-0.76	0.1356	-1.65	0.0102	hypothetical protein	sufD, sufB	P	
slr1623	1.02	0.0139	-0.63	0.0620	-1.32	0.0016	hypothetical protein	ndhM	P	
sll1455	1.01	0.0261	0.20	0.2634	-0.10	0.7055	hypothetical protein	narM	P	
ssr3410	0.07	0.7826	-1.64	0.0021	-1.44	0.0027	hypothetical protein	mrpG	P	
slr2008	0.05	0.8490	-1.90	0.0004	-1.63	0.0015	hypothetical protein	mrpC	P	

ORF	ΔrpoZ$_{40}$/ΔrpoZ$_{32}$		CS$_{40}$/CS$_{32}$		ΔrpoZ$_{32}$/CS$_{32}$		Description	Gene name	Functional category**
	FC*	P-value	FC*	P-value	FC*	P-value			
slr2006	0.43	0.1619	-3.63	0.0012	-3.36	0.0000	hypothetical protein	mrpC	P
slr2011	0.14	0.5252	-1.73	0.0020	-1.64	0.0013	hypothetical protein	mrpA	P
slr1152	-0.38	0.2282	2.08	0.0011	2.33	0.0011	hypothetical protein	rfrK	P
ssr3409	0.00	0.9867	-1.58	0.0008	-1.48	0.0016	hypothetical protein	mrpF	P
slr2010	-0.04	0.9014	-1.96	0.0016	-1.65	0.0040	hypothetical protein	mrpE	P
slr2012	-0.27	0.1391	-1.58	0.0010	-1.27	0.0037	hypothetical protein	mrpB	P
sll1473	-1.01	0.0023	-0.06	0.7857	0.88	0.0089	a part of phytochrome-like sensor histidine kinase gene (disrupted by insertion of IS)	hik32	P
slr0359	-1.05	0.0079	0.21	0.1338	1.01	0.0007	hypothetical protein	syn-lov	P
sll0905	-1.13	0.0011	-0.11	0.4924	-0.31	0.0348	hypothetical protein	maf	P
slr2031	-1.23	0.0026	0.14	0.4768	1.47	0.0005	putative PP2C-type protein phosphatase, gene required to recover from the nitrogen or sulfate starvation induced stationary phase	rsbU	P
sll1358	-1.32	0.0001	-0.46	0.0033	0.96	0.0001	putative oxalate decarboxylase, periplasmic protein	mncA	P
slr1840	-1.38	0.0040	-0.12	0.7435	0.45	0.2143	hypothetical protein	synGK	P
slr0244	-1.42	0.0001	0.32	0.0042	1.12	0.0001	hypothetical protein	usp	P
slr0148	-1.49	0.0001	0.34	0.2550	1.72	0.0014	hypothetical protein	fdx	P
sll1654	-1.92	0.0003	0.52	0.1059	2.15	0.0000	hypothetical protein	usp	P
slr1963	-2.01	0.0004	-0.48	0.0277	-0.10	0.6185	water-soluble carotenoid protein	ocp	P
slr1851	-2.51	0.0018	0.10	0.6728	3.74	0.0001	hypothetical protein	rfrH	P
ssl1911	-6.29	0.0000	-1.25	0.2978	0.97	0.0149	glutamine synthetase inactivating factor IF7	gifA	P
sll1158	7.90	0.0000	3.42	0.0000	-2.65	0.0001	hypothetical protein		P
sll1160	4.17	0.0001	2.07	0.0000	-0.74	0.0335	hypothetical protein		P
sll0787	2.85	0.0004	1.40	0.1299	-0.05	0.8609	hypothetical protein		P
slr0292	2.48	0.0002	0.36	0.2201	-1.08	0.0003	hypothetical protein		P
slr1770	2.34	0.0000	1.16	0.0374	-0.46	0.0322	hypothetical protein		P
sll1119	2.21	0.0011	2.65	0.0147	0.70	0.2812	hypothetical protein		P
slr1220	1.95	0.0166	-0.32	0.2370	-1.96	0.0062	hypothetical protein		P
sll1251	1.84	0.0004	0.05	0.9086	0.00	0.9874	hypothetical protein		P
slr1906	1.68	0.0024	0.53	0.1172	-1.32	0.0072	hypothetical protein		P
sll0549	1.64	0.0000	-0.16	0.4106	-0.77	0.0032	hypothetical protein		P
slr0300	1.61	0.0017	0.64	0.0337	0.07	0.6823	hypothetical protein		P
ssl1762	1.57	0.0034	1.21	0.0433	0.67	0.0954	hypothetical protein		P
slr1119	1.55	0.0005	0.43	0.4159	-0.61	0.0136	hypothetical protein		P
slr1612	1.54	0.0101	-0.03	0.9336	-0.03	0.9345	hypothetical protein		P
sll1783	1.47	0.0003	0.57	0.0028	-0.25	0.1065	hypothetical protein		P
sll0846	1.41	0.0102	0.79	0.0642	0.82	0.0310	hypothetical protein		P
slr1674	1.40	0.0004	0.72	0.1486	0.75	0.0065	hypothetical protein		P
sll0498	1.39	0.0001	0.85	0.0083	0.53	0.0217	hypothetical protein		P
sll1080	1.39	0.0008	0.93	0.1781	0.92	0.0031	ABC transport system substrate-binding protein		P
sll0261	1.36	0.0006	1.20	0.0647	0.88	0.0013	hypothetical protein		P
sll1162	1.36	0.0008	0.95	0.0357	0.11	0.6175	hypothetical protein		P
slr1611	1.30	0.0011	0.05	0.8735	0.38	0.0650	hypothetical protein		P
ssr0550	1.28	0.0096	0.41	0.0351	-1.08	0.0094	hypothetical protein		P

ORF	ΔrpoZ₄₀/ΔrpoZ₃₂		CS₄₀/CS₃₂		ΔrpoZ₃₂/CS₃₂		Description	Gene name	Functional category**
	FC*	P-value	FC*	P-value	FC*	P-value			
sll0837	1.27	0.0407	0.07	0.5629	-0.61	0.1602	periplasmic protein, function unknown		P
sll0888	1.26	0.0023	0.64	0.0001	0.66	0.0047	hypothetical protein		P
slr0751	1.25	0.0013	0.61	0.0001	-0.22	0.1182	hypothetical protein		P
sll1039	1.24	0.0021	1.22	0.0546	0.23	0.2774	hypothetical protein		P
slr0964	1.23	0.0289	-0.12	0.6104	-1.25	0.0160	hypothetical protein		P
slr1376	1.19	0.0150	0.64	0.0018	0.05	0.8342	hypothetical protein		P
sll0218	1.15	0.0090	-1.09	0.5165	-4.75	0.0005	hypothetical protein		P
slr0320	1.14	0.0004	-0.80	0.0077	-1.74	0.0001	hypothetical protein		P
slr1799	1.13	0.0009	-0.19	0.5823	-1.10	0.0045	hypothetical protein		P
slr0397	1.12	0.0417	0.46	0.1030	-0.46	0.1440	hypothetical protein		P
sll0983	1.11	0.0076	0.14	0.5557	-0.03	0.9009	hypothetical protein		P
sll0944	1.10	0.0372	1.25	0.0280	0.57	0.2138	hypothetical protein		P
slr2125	1.09	0.0067	0.05	0.8255	-0.73	0.0324	hypothetical protein		P
slr1660	1.09	0.0089	0.39	0.0016	0.03	0.8621	hypothetical protein		P
ssl0352	1.07	0.0042	-0.78	0.0171	-1.09	0.0015	hypothetical protein		P
slr1717	1.06	0.0000	0.17	0.5298	-0.23	0.0079	hypothetical protein		P
sll0812	1.05	0.0012	0.75	0.0011	0.08	0.4392	hypothetical protein		P
slr1753	1.04	0.0098	0.14	0.4216	-1.14	0.0025	hypothetical protein		P
slr0769	1.04	0.0017	-0.02	0.9444	-0.58	0.0036	hypothetical protein		P
sll0036	1.01	0.0447	-0.06	0.4306	-0.63	0.0747	hypothetical protein		P
slr1290	1.01	0.0010	1.05	0.0236	0.92	0.0001	hypothetical protein		P
sll0335	0.72	0.0373	1.62	0.0222	1.11	0.0114	hypothetical protein		P
sll1696	0.60	0.0128	1.41	0.0001	0.56	0.0065	hypothetical protein		P
ssl0331	0.17	0.6186	1.12	0.0350	0.79	0.1092	hypothetical protein		P
slr1913	0.51	0.1470	1.01	0.0020	0.26	0.1528	hypothetical protein		P
sll0822	0.18	0.0879	-1.06	0.0122	-1.38	0.0003	hypothetical protein		P
slr1472	-0.45	0.0501	-1.06	0.0075	-0.63	0.0619	hypothetical protein		P
slr0870	-0.49	0.3779	-1.07	0.0008	-1.33	0.0187	hypothetical protein		P
slr1677	0.30	0.2791	-1.07	0.0371	-0.77	0.1024	hypothetical protein		P
sll1911	-0.30	0.4877	-1.14	0.0218	-1.00	0.0525	hypothetical protein		P
sll0529	0.65	0.0655	-1.17	0.0295	-2.14	0.0001	hypothetical protein		P
ssl2874	0.81	0.0816	-1.23	0.0011	-2.51	0.0003	hypothetical protein		P
slr2013	0.10	0.6659	-1.25	0.0007	-1.10	0.0042	hypothetical protein		P
ssl2921	0.26	0.4812	-1.40	0.0310	-0.73	0.1475	hypothetical protein		P
ssl2920	0.03	0.9667	-1.53	0.0232	-0.66	0.4167	hypothetical protein		P
sll1504	0.05	0.9328	-1.57	0.0014	-0.65	0.2083	hypothetical protein		P
sll1505	0.37	0.6854	-1.94	0.0145	-0.89	0.3230	hypothetical protein		P
sll0185	-0.33	0.0229	-1.60	0.0342	-0.18	0.4638	hypothetical protein		P
sll1469	-0.38	0.0135	1.02	0.0010	0.84	0.0022	hypothetical protein		P
ssr2998	-0.72	0.0221	-1.02	0.0077	-0.15	0.5957	hypothetical protein		P
sll1483	-0.81	0.0018	-1.04	0.0031	-0.15	0.4310	periplasmic protein, similar to transforming growth factor induced protein		P
slr0878	-1.01	0.0011	-0.24	0.3568	0.44	0.0362	hypothetical protein		P
slr0686	-1.01	0.0049	-0.34	0.0552	0.73	0.0135	hypothetical protein		P
slr0249	-1.01	0.0012	0.56	0.0069	1.46	0.0001	hypothetical protein		P

ORF	ΔrpoZ$_{40}$/ΔrpoZ$_{32}$		CS$_{40}$/CS$_{32}$		ΔrpoZ$_{32}$/CS$_{32}$		Description	Gene name	Functional category**
	FC*	P-value	FC*	P-value	FC*	P-value			
slr0105	-1.03	0.0110	0.01	0.9796	-1.00	0.0166	hypothetical protein		P
slr0053	-1.03	0.0058	-0.66	0.0362	0.52	0.0003	hypothetical protein		P
slr1241	-1.05	0.0431	0.01	0.9387	0.86	0.0320	hypothetical protein		P
ssr2802	-1.05	0.0151	1.01	0.0562	1.31	0.0155	hypothetical protein		P
slr1161	-1.11	0.0110	-0.02	0.8443	-0.02	0.8864	hypothetical protein		P
sll0360	-1.12	0.0036	-1.00	0.0148	-0.11	0.6549	hypothetical protein		P
slr1444	-1.13	0.0001	-0.60	0.0111	0.55	0.0045	hypothetical protein		P
slr1692	-1.14	0.0029	-1.06	0.0294	0.59	0.1568	hypothetical protein		P
sll0023	-1.15	0.0159	0.39	0.3243	1.14	0.0089	hypothetical protein		P
sll0611	-1.16	0.0003	-0.30	0.3373	0.63	0.0581	hypothetical protein		P
sll0615	-1.18	0.0092	-0.42	0.0171	0.07	0.6455	hypothetical protein		P
sll1965	-1.23	0.0044	-0.81	0.0145	0.77	0.0110	hypothetical protein		P
slr0554	-1.23	0.0014	-0.92	0.0002	0.05	0.6801	hypothetical protein		P
sll1348	-1.26	0.0004	-0.49	0.1578	-0.09	0.7653	hypothetical protein		P
slr1506	-1.30	0.0193	-0.38	0.0040	0.28	0.3549	hypothetical protein		P
slr0959	-1.33	0.0009	-1.68	0.0009	0.57	0.0669	hypothetical protein		P
ssl1046	-1.35	0.0003	-0.34	0.1070	1.06	0.0010	hypothetical protein		P
sll0412	-1.35	0.0018	0.21	0.1989	1.63	0.0002	hypothetical protein		P
slr2052	-1.37	0.0021	-0.08	0.8585	1.40	0.0019	hypothetical protein		P
sll0543	-1.39	0.0002	-0.41	0.0836	1.06	0.0017	hypothetical protein		P
slr0179	-1.40	0.0068	0.12	0.7699	0.33	0.4378	hypothetical protein		P
ssl3573	-1.43	0.0025	0.06	0.8008	2.01	0.0006	hypothetical protein		P
slr0869	-1.45	0.0168	-0.27	0.4341	0.03	0.9097	hypothetical protein		P
slr1971	-1.46	0.0026	0.11	0.7467	0.63	0.0348	hypothetical protein		P
slr1068	-1.47	0.0058	-0.79	0.0356	-0.04	0.8585	hypothetical protein		P
slr0146	-1.47	0.0060	1.03	0.0015	2.65	0.0001	hypothetical protein		P
sll1516	-1.52	0.0017	-1.18	0.0049	0.16	0.5648	hypothetical protein		P
slr0789	-1.63	0.0003	-1.28	0.0568	-0.64	0.0145	hypothetical protein		P
slr1069	-1.64	0.0173	-1.63	0.0003	0.57	0.0553	hypothetical protein		P
slr1260	-1.67	0.0001	-0.54	0.0208	0.85	0.0019	hypothetical protein		P
sll0572	-1.70	0.0196	0.02	0.9364	1.26	0.0330	hypothetical protein		P
sll0051	-1.70	0.0010	-0.20	0.4931	0.94	0.0258	hypothetical protein		P
sll1500	-1.75	0.0068	-0.26	0.1432	1.33	0.0081	hypothetical protein		P
slr0147	-1.77	0.0000	0.75	0.0269	2.45	0.0002	hypothetical protein		P
ssr2062	-1.79	0.0002	-1.27	0.0038	0.33	0.1497	hypothetical protein		P
slr1957	-1.87	0.0025	0.59	0.1358	2.20	0.0009	hypothetical protein		P
sll1722	-1.94	0.0258	-1.41	0.0926	0.52	0.5194	hypothetical protein		P
sll0283	-1.96	0.0001	0.14	0.5332	2.31	0.0000	hypothetical protein		P
slr1259	-2.00	0.0001	-0.39	0.0435	1.26	0.0002	hypothetical protein		P
slr0680	-2.12	0.0014	0.04	0.8090	2.16	0.0002	hypothetical protein		P
slr0144	-2.25	0.0009	1.14	0.0006	3.83	0.0000	hypothetical protein		P
ssr1562	-2.32	0.0003	-0.61	0.4092	0.85	0.0185	hypothetical protein		P
slr1438	-2.41	0.0001	-0.03	0.9230	2.84	0.0000	hypothetical protein		P
sll0451	-2.61	0.0005	-1.15	0.0142	0.86	0.0361	hypothetical protein		P
sll0253	-2.85	0.0003	0.21	0.4392	2.45	0.0005	hypothetical protein		P
ssr1251	-3.30	0.0002	-1.43	0.0173	1.74	0.0019	hypothetical protein		P

ORF	ΔrpoZ40/ΔrpoZ32		CS40/CS32		ΔrpoZ32/CS32		Description	Gene name	Functional category**	
	FC*	P-value	FC*	P-value	FC*	P-value				
slr0888	-3.42	0.0001	0.49	0.2391	3.11	0.0003	hypothetical protein		P	
slr1704	-3.86	0.0000	-0.41	0.4343	3.56	0.0004	hypothetical protein		P	
ssr0692	-3.94	0.0000	-1.80	0.1120	0.72	0.0157	hypothetical protein		P	
sll1293	1.29	0.0123	0.53	0.0028	-0.84	0.0185	unknown protein	taxW2	Z	Unknown
sll1785	1.06	0.0010	0.51	0.0356	-0.09	0.3996	periplasmic protein, function unknown	cucA	Z	
sll1583	-1.05	0.0079	0.46	0.0347	1.67	0.0000	unknown protein	ligA	Z	
slr0593	-1.12	0.0457	0.12	0.7443	0.72	0.0655	cAMP binding membrane protein	samp	Z	
sll1009	-1.84	0.0017	-0.46	0.1438	-0.23	0.4961	unknown protein	frpC	Z	
slr0616	-1.86	0.0005	-2.44	0.1374	-3.19	0.0000	unknown protein	mvrA	Z	
slr1667	-2.05	0.0271	0.49	0.0014	2.82	0.0026	hypothetical protein (target gene of sycrp1)	cccS	Z	
sll1515	-5.02	0.0000	-1.86	0.0838	0.72	0.1089	glutamine synthetase inactivating factor IF17	gifB	Z	
ssl2814	4.86	0.0000	1.13	0.3497	-2.53	0.0013	unknown protein		Z	
sll0783	4.55	0.0001	3.84	0.0276	1.46	0.0071	unknown protein		Z	
sll0786	3.01	0.0005	1.90	0.1120	0.13	0.4947	unknown protein		Z	
sll0785	2.92	0.0021	2.22	0.0753	0.75	0.0775	unknown protein		Z	
slr1852	2.46	0.0003	0.71	0.0629	-0.54	0.1064	unknown protein		Z	
sll0733	2.06	0.0097	2.06	0.0112	0.74	0.2204	unknown protein		Z	
ssl3410	2.01	0.0000	0.49	0.0415	-0.88	0.0022	unknown protein		Z	
slr1854	1.96	0.0019	0.19	0.4553	-0.83	0.0209	unknown protein		Z	
slr1855	1.95	0.0003	-0.04	0.8325	-1.35	0.0013	unknown protein		Z	
sll1784	1.86	0.0001	0.51	0.0350	-0.61	0.0033	periplasmic protein, function unknown		Z	
ssl2507	1.77	0.0018	1.26	0.0750	-0.32	0.1831	unknown protein		Z	
sll1304	1.64	0.0003	0.11	0.6757	-0.79	0.0233	unknown protein		Z	
sll0293	1.63	0.0001	0.73	0.0291	-0.54	0.0326	unknown protein		Z	
ssl2501	1.49	0.0027	0.76	0.2065	0.44	0.1013	unknown protein		Z	
sll1163	1.49	0.0012	0.82	0.0963	0.24	0.4480	unknown protein		Z	
ssl2502	1.42	0.0036	0.80	0.0664	-0.31	0.1419	unknown protein		Z	
sll1396	1.40	0.0007	0.97	0.0145	1.07	0.0011	unknown protein		Z	
sll0188	1.39	0.0020	0.66	0.1313	0.37	0.3247	unknown protein		Z	
slr1135	1.24	0.0113	-1.18	0.1178	-1.29	0.0890	unknown protein		Z	
sll0225	1.20	0.0023	0.76	0.0387	-0.42	0.1037	unknown protein		Z	
slr0168	1.18	0.0044	-0.13	0.7591	-1.16	0.0107	unknown protein		Z	
ssl1464	1.16	0.0173	1.01	0.1103	0.82	0.0245	unknown protein		Z	
slr2018	1.11	0.0031	0.16	0.5248	-1.05	0.0018	unknown protein		Z	
sll1306	1.11	0.0010	-0.50	0.1308	-1.05	0.0047	periplasmic protein, function unknown		Z	
slr0273	1.10	0.0089	0.86	0.0070	-0.30	0.3156	unknown protein		Z	
slr0617	1.08	0.0366	0.88	0.0004	0.02	0.9617	unknown protein		Z	
sll0982	1.08	0.0059	0.32	0.3097	0.30	0.1798	unknown protein		Z	
sll0172	1.07	0.0058	1.24	0.0513	0.67	0.0109	periplasmic protein, function unknown		Z	
slr0345	1.03	0.0002	0.41	0.0206	-0.97	0.0004	unknown protein		Z	
ssl1493	1.01	0.0002	0.23	0.0698	-0.28	0.0021	unknown protein		Z	
slr1484	0.95	0.0002	1.80	0.0016	0.27	0.3502	unknown protein		Z	
slr0442	0.90	0.0003	1.29	0.0052	0.47	0.0528	unknown protein		Z	

ORF	ΔrpoZ$_{40}$/ΔrpoZ$_{32}$		CS$_{40}$/CS$_{32}$		ΔrpoZ$_{32}$/CS$_{32}$		Description	Gene name	Functional category**
	FC*	P-value	FC*	P-value	FC*	P-value			
sll0441	0.45	0.1085	1.35	0.0118	1.70	0.0018	unknown protein		Z
slr1681	-0.04	0.8890	1.17	0.0210	1.09	0.0176	unknown protein		Z
sll0327	-0.52	0.1545	1.13	0.0482	0.02	0.9762	unknown protein		Z
slr1920	-0.22	0.1932	1.10	0.0266	0.07	0.6473	unknown protein		Z
slr0262	0.04	0.7640	-1.03	0.0208	-1.19	0.0002	unknown protein		Z
slr1618	0.08	0.8421	-1.06	0.0027	-0.05	0.8890	unknown protein		Z
slr0708	0.22	0.4614	-1.10	0.0102	-0.94	0.0078	periplasmic protein, function unknown		Z
sll0710	-0.42	0.4358	-1.30	0.0004	-0.52	0.2313	unknown protein		Z
slr1066	-0.37	0.0578	-1.33	0.0038	-0.25	0.3370	unknown protein		Z
slr0871	-0.24	0.5978	-1.47	0.0091	-1.81	0.0059	unknown protein		Z
sll0263	-0.12	0.8078	-1.54	0.0052	-2.01	0.0068	unknown protein		Z
slr1726	-0.82	0.0164	1.06	0.0014	1.03	0.0011	unknown protein		Z
slr1383	-0.94	0.0018	-1.18	0.0251	-0.80	0.0139	unknown protein		Z
sll1611	-0.99	0.0023	-1.11	0.0099	-0.44	0.1963	unknown protein		Z
sll0723	-1.02	0.0055	0.23	0.2136	0.70	0.0143	unknown protein		Z
sll0444	-1.02	0.0004	0.21	0.2607	0.84	0.0029	unknown protein		Z
slr1258	-1.03	0.0319	-0.04	0.8126	0.86	0.0331	unknown protein		Z
ssl1326	-1.04	0.0058	-0.54	0.2505	0.50	0.1424	unknown protein		Z
slr1788	-1.08	0.0138	-0.18	0.1320	-2.30	0.0000	unknown protein		Z
slr0868	-1.09	0.0056	-0.58	0.0824	-0.47	0.1823	unknown protein		Z
slr0582	-1.10	0.0010	-0.10	0.4712	1.28	0.0003	unknown protein		Z
slr0069	-1.11	0.0014	0.20	0.3658	0.86	0.0021	unknown protein		Z
slr1073	-1.16	0.0132	-1.40	0.0000	-0.24	0.3309	unknown protein		Z
ssr0693	-1.17	0.0096	0.50	0.0133	1.86	0.0005	unknown protein		Z
ssl1533	-1.19	0.0010	-0.55	0.0606	0.93	0.0060	unknown protein		Z
sll1882	-1.21	0.0029	0.02	0.9159	1.31	0.0015	unknown protein		Z
slr1074	-1.21	0.0197	-1.58	0.0004	-0.35	0.2502	unknown protein		Z
sll1429	-1.27	0.0483	0.21	0.6987	0.67	0.1688	unknown protein		Z
sll0376	-1.27	0.0062	0.99	0.0034	2.81	0.0001	unknown protein		Z
slr1071	-1.28	0.0001	-1.59	0.0001	-0.08	0.4285	unknown protein		Z
ssl2653	-1.38	0.0015	-1.33	0.0533	0.52	0.0514	unknown protein		Z
ssl2891	-1.43	0.0156	0.00	0.9853	1.95	0.0017	unknown protein		Z
slr1544	-1.50	0.0017	-0.92	0.0752	0.36	0.1684	unknown protein		Z
slr1070	-1.54	0.0028	-0.70	0.0185	0.60	0.0168	unknown protein		Z
slr1670	-1.56	0.0016	-0.62	0.0096	1.44	0.0001	unknown protein		Z
ssl0318	-1.57	0.0072	0.29	0.3291	1.74	0.0008	unknown protein		Z
slr1958	-1.57	0.0039	0.16	0.7587	2.15	0.0004	unknown protein		Z
slr0226	-1.64	0.0062	0.38	0.1114	2.79	0.0002	unknown protein		Z
slr0579	-1.66	0.0014	0.13	0.6766	2.13	0.0005	unknown protein		Z
ssr2153	-1.66	0.0052	-1.42	0.0040	1.28	0.0045	unknown protein		Z
sll0775	-1.82	0.0035	-0.37	0.4073	-1.24	0.0205	unknown protein		Z
sll1236	-1.85	0.0017	-0.09	0.4476	2.07	0.0002	unknown protein		Z
ssl2384	-1.86	0.0005	2.15	0.0002	4.32	0.0000	unknown protein		Z
slr0145	-1.90	0.0006	1.14	0.0005	3.36	0.0000	unknown protein		Z
slr0587	-2.09	0.0006	-0.45	0.0169	2.27	0.0001	unknown protein		Z

ORF	ΔrpoZ40/ΔrpoZ32		CS40/CS32		ΔrpoZ32/CS32		Description	Gene name	Functional category**
	FC*	P-value	FC*	P-value	FC*	P-value			
slr0581	-2.36	0.0001	-0.63	0.1499	1.21	0.0019	unknown protein		Z
ssr3129	-2.56	0.0019	0.14	0.5404	3.76	0.0001	unknown protein		Z
slr1862	-2.61	0.0058	-0.01	0.9702	3.62	0.0004	unknown protein		Z
slr1437	-2.74	0.0001	-0.09	0.6212	3.35	0.0000	unknown protein		Z
sll1241	-3.17	0.0002	-1.68	0.0099	1.01	0.0344	unknown protein		Z
sll1239	-3.33	0.0001	-1.40	0.0371	0.81	0.0946	unknown protein		Z
ssr2194	-3.77	0.0001	-1.92	0.0011	3.61	0.0001	unknown protein		Z
sll1240	-3.84	0.0013	-1.81	0.0893	1.04	0.0765	unknown protein		Z
ssr1038	-4.72	0.0001	-1.40	0.2528	0.57	0.0379	unknown protein		Z

*FC: $\log_2$ of fold change.
**The categories are listed according to Cyanobase.
