# Supplementary material for: The ω Subunit of RNA Polymerase Is Essential for Thermal Acclimation of the Cyanobacterium Synechocystis Sp. PCC 6803
Source: PLoS One. 2014 Nov 11;9(11):e112599. doi: 10.1371/journal.pone.0112599 (PMC4227741; doi:10.1371/journal.pone.0112599)
Supplement: Table S6 — List of genes that were similarly or oppositely regulated in CS and ΔrpoZ after heat treatment. (PDF) [file pone.0112599.s006.pdf]

Table S6. List of genes that were similarly or oppositely regulated in CS and ΔrpoZ after heat treatment.

ORF	CS_{40}/CS_{32}		$\Delta rpoZ_{40}/\Delta rpoZ_{32}$		Function	Gene
	FC*	P value	FC*	P value		
sll0450	-2.39	1.77E-04	-3.10	3.30E-05	cytochrome b subunit of nitric oxide reductase	*norB*
slr0426	-1.02	4.05E-02	-1.37	2.66E-04	GTP cyclohydrolase I	*folE*
slr0749	-1.11	4.19E-03	-2.84	3.00E-06	light-independent protochlorophyllide reductase iron protein subunit ChlL	*chlL*
slr1072	-1.11	9.30E-05	-1.05	7.09E-04	GDP-D-mannose dehydratase	*rfbD, yefA*
slr1351	-1.01	4.15E-02	-1.51	1.74E-03	UDP-N-acetylmuramoylalanyl-D-glutamyl-2 6-diaminopimelate--D-alanyl-D-alanine ligase	*murF*
slr2075	-1.08	1.06E-02	-1.86	1.41E-04	10kD chaperonin	*groES*
slr0427	-1.00	2.35E-03	-1.16	8.43E-04	putative competence-damage protein	*psbA2*
slr1705	-1.26	3.77E-03	-2.02	4.38E-03	aspartoacylase	*aspA*
sll1441	-2.40	1.89E-03	-1.34	3.03E-02	acyl-lipid desaturase (omega-3)	*desB*
sll0330	-1.19	4.70E-02	-1.01	5.10E-03	sepiapterine reductase	*fabG*
ssl3044	-1.23	6.94E-03	-1.71	1.90E-02	probable ferredoxin	
slr1164	-1.41	7.00E-06	-1.27	6.40E-04	ribonucleotide reductase subunit alpha	*nrdA, dnaF*
slr1214	-1.09	1.43E-02	-1.98	3.26E-03	two-component response regulator	*rre15*
sll1626	-1.33	8.20E-03	-1.81	4.59E-04	LexA repressor	*lexA*
slr0790	-1.47	4.40E-02	-1.45	2.72E-04	similar to ultraviolet light resistance protein B	*umuC*
slr1019	-1.49	4.54E-02	-1.40	9.61E-04	phenazine biosynthetic protein PhzF homolog	
sll0222	-1.04	1.55E-02	-1.08	6.27E-03	putative purple acid phosphatase	*phoA*
slr1065	-1.49	2.57E-04	-1.06	9.09E-03	probable glycosyltransferase	
sll0360	-1.00	1.48E-02	-1.12	3.56E-03	hypothetical protein	
sll1516	-1.18	4.88E-03	-1.52	1.74E-03	hypothetical protein	
ssr2062	-1.27	3.77E-03	-1.79	1.56E-04	hypothetical protein	
slr0959	-1.68	9.08E-04	-1.33	8.68E-04	hypothetical protein	
slr1069	-1.63	2.52E-04	-1.64	1.73E-02	hypothetical protein	
slr1692	-1.06	2.94E-02	-1.14	2.87E-03	hypothetical protein	
sll0451	-1.15	1.42E-02	-2.61	5.06E-04	hypothetical protein	
ssr1251	-1.43	1.73E-02	-3.30	1.56E-04	hypothetical protein	
slr1074	-1.58	4.15E-04	-1.21	1.97E-02	unknown protein	
slr1073	-1.40	1.40E-05	-1.16	1.32E-02	unknown protein	
slr1071	-1.59	8.40E-05	-1.28	1.15E-04	unknown protein	
sll1239	-1.40	3.71E-02	-3.33	1.46E-04	unknown protein	
sll1241	-1.68	9.91E-03	-3.17	2.06E-04	unknown protein	
ssr2153	-1.42	4.03E-03	-1.66	5.20E-03	unknown protein	
ssr2194	-1.92	1.12E-03	-3.77	1.05E-04	unknown protein	
slr0146	1.03	1.52E-03	-1.47	5.99E-03	hypothetical protein	
slr0144	1.14	6.28E-04	-2.25	8.73E-04	hypothetical protein	
ssl2384	2.15	2.29E-04	-1.86	4.70E-04	unknown protein	
sll1198	1.73	4.00E-03	-1.79	3.73E-03	tRNA (guanine-N1)-methyltransferase	*trmD*
slr0145	1.14	4.59E-04	-1.90	5.92E-04	unknown protein	
slr0293	-1.17	7.47E-04	1.71	2.17E-03	glycine dehydrogenase	*gcvP*
sll1594	-3.11	3.01E-04	1.53	3.80E-02	ndhF3 operon transcriptional regulator, LysR	*ccmR, ndhR*

ORF	CS_{40}/CS_{32}		$\Delta rpoZ_{40}/\Delta rpoZ_{32}$		Function	Gene
	FC*	P value	FC*	P value		
					family protein	
slr1756	2.01	1.27E-02	1.79	1.50E-05	glutamate--ammonia ligase	*glnA*
slr0288	2.65	4.23E-02	1.79	3.83E-04	glutamate--ammonia ligase	*glnN*
slr1289	1.81	9.67E-03	1.77	1.97E-04	isocitrate dehydrogenase (NADP+)	*icdA*
ssl2559	1.23	2.32E-03	2.26	4.07E-03	ferredoxin	
slr0851	1.20	3.50E-02	1.31	7.00E-04	type 2 NADH dehydrogenase	*ndbA*
sll1161	1.71	3.43E-04	4.55	1.07E-04	probable adenylate cyclase	*cya3*
sll0782	1.94	2.04E-02	2.00	1.16E-02	transcriptional regulator	
sll1330	1.83	1.97E-02	1.40	1.22E-02	two-component system response regulator OmpR subfamily	*rre37*
sll2008	1.08	1.54E-02	1.78	2.46E-04	processing protease	*prp1*
sll1270	1.95	1.58E-02	1.98	1.21E-02	periplasmic substrate-binding and integral membrane protein of the ABC-type Bgt permease for basic amino acids and glutamine	*bgtB*
sll1017	2.17	3.62E-02	1.30	2.22E-04	ammonium/methylammonium permease	*amt2*
sll0108	2.23	2.16E-02	2.08	4.69E-04	ammonium/methylammonium permease	*amt1*
slr0944	1.03	2.81E-02	1.91	4.28E-04	multidrug-efflux transporter	*arsB*
slr1735	1.13	3.68E-02	1.39	7.27E-04	ATP-binding subunit of the ABC-type Bgt permease for basic amino acids and glutamine	*bgtA*
slr1200	3.12	5.60E-03	1.51	4.53E-04	urea transport system permease protein	*urtB*
sll1599	1.63	1.50E-02	1.47	1.94E-04	manganese transport system ATP-binding protein MntA	*mntA*
sll0536	2.09	1.50E-02	1.36	8.09E-03	probable potassium channel protein	*kchX*
sll1598	1.88	1.16E-02	1.70	1.11E-04	Mn transporter MntC	*mntC*
sll1159	2.86	3.00E-06	6.08	3.00E-05	probable bacterioferritin comigratory protein	
slr2002	1.66	3.69E-02	1.65	4.53E-03	cyanophycin synthetase	*cphA*
sll1158	3.42	1.00E-06	7.90	3.00E-06	hypothetical protein	
sll1160	2.07	3.60E-05	4.17	1.34E-04	hypothetical protein	
slr1770	1.16	3.74E-02	2.34	5.00E-06	hypothetical protein	
sll0944	1.25	2.80E-02	1.10	3.72E-02	hypothetical protein	
ssl1762	1.21	4.33E-02	1.57	3.40E-03	hypothetical protein	
sll1119	2.65	1.47E-02	2.21	1.10E-03	hypothetical protein	
slr1290	1.05	2.36E-02	1.01	1.03E-03	hypothetical protein	
sll0733	2.06	1.12E-02	2.06	9.73E-03	unknown protein	
sll0783	3.84	2.76E-02	4.55	7.00E-05	unknown protein	

*FC: $\log_2$ of fold change
